# Acknowledgment to Reviewers of *Plants* in 2021

**DOI:** 10.3390/plants11040470

**Published:** 2022-02-09

**Authors:** Plants Editorial Office

**Affiliations:** MDPI AG, St. Alban-Anlage 66, 4052 Basel, Switzerland

Rigorous peer-reviews are the basis of high-quality academic publishing. Thanks to the great efforts of our reviewers, *Plants* was able to maintain its standards for the high quality of its published papers. Thanks to the contribution of our reviewers, in 2021, the median time to first decision was 13 days and the median time to publication was 34 days. The editors would like to extend their gratitude and recognition to the following reviewers for their precious time and dedication, regardless of whether the papers they reviewed were finally published:
Abate, GiuliaLamport, Derek T. A.Abbate, CristinaLandi, MarcoAbbate, LoredanaLangat, MosesAbdallah, Mohamed FathiLanghansova, LenkaAbdel Latef, Arafat Abdel HamedLangridge, PeterAbedini-Nassab, RoozbehLannacone, RinaAbouelenein, DoaaLanoue, JasonAbrantes, IsabelLantos, CsabaAbubakar, AliyuLapidot, MosheAccoroni, StefanoLargo-Gosens, AsierAcedo, CarmenLarkin, John C.Acosta Motos, Jose RamónLarriba Tornel, EduardoAcquaviva, RosariaLarson, Steven R.Ádám, Attila L.Lastochkina, OksanaAdamakis, Ioannis-DimosthenisLászló Orbán, CsambalikAdamczewska-Sowińska, KatarzynaLászló, ErdeiAdamus, AdelaLatowski, DariuszAdhikari, BishwoLatté, Klaus PeterAdil, MuhammadLattorff, H. Michael G.Adkar-Purushothama, Charith RajLauberts, MarisAdnan, Md.Lauceri, RosariaAdriana, PauceanLaureys, DavidAfonnikov, Dimitry ArkadievichLaval-Gilly, PhilippeAfonso, Andrea Luísa FernandesLavkulich, Les M.Agliassa, ChiaraLavric, VasileAgudelo-Romero, PatriciaLaw, Henry Chun HinAguiar, Francisca C.Laws, Edward A.Ahanger, Mohammad AbassLazare, SilitAhmad, KhurshidLazarević, BorisAhmad, MargaretLazarovits, GeorgeAhmad, NiazLe Signor, ChristineAhmed Malik, OwaisLe, Phuong MaiAhmed, EzzatLeahu, AnaAhmed, IshtiaqLean, DavidAhmed, MukhtarLeborgne-Castel, NathalieAide, Michael T.Lechowicz, KatarzynaAigotti, RiccardoLecomte, PascalAkama, KazuhitoLee, Hwa-JinAkihiro, TakashiLee, HyeyoungAkkak, AzizLee, HyohyemiAksmann, AnnaLee, Hyun-sookAl Bari, Md. AbdullahLee, JaehyuckAlam, MobashwerLee, Jin-KooAlamillo, Josefa MuñozLee, JiyoungAlarcon, JulioLee, Jong HeeAl-Ashkar, IbrahimLee, Jung-HyunAlbuquerque-Júnior, Ricardo Luiz Cavalcanti DeLee, JunsooAlburquerque, NuriaLee, KwanukAlcántara-de la Cruz, RicardoLee, Kyeong-YeollAleffi, MicheleLee, Kyung JunAlejandro, SantiagoLee, Marcia R.Aleksandrowicz-Trzcińska, MartaLee, MinaAlepuz, PaulaLee, NatuschkaAletà, NeusLee, RichardAlexandrov, Oleg SergeevichLee, Sang JunAlexopoulos, Alexios A.Lee, Sang-HanAleynova, Olga A.Lee, Sung WooAl-Fatimi, MohamedLee, SungbeomAli, AkhtarLeggewie, GeorgAli, AnwarLeglize, PierreAli, ImranLeisova-Svobodova, LeonaAli, NusratLeitão, Susana TrindadeAli, SajadLejman, AgnieszkaAlías-Gallego, Juan CarlosLemic, DarijaAlicja, Macko-PodgorniLemieszek, MartaAlicja, TymoszukLemli, BeátaAlkowni, RaedLeon, FranciscoAllaby, RobinLeon, JensAllegrezza, MarinaLeón-Félix, JosefinaAllen, Edith B.Leonova, IrinaAlmeida Machado, Rui ManuelLeón-Rivera, IsmaelAlok, AnshuLeontina, LipanAlov, PetkoLeontopoulos, StefanosAlptekin, BurcuLepinay, ClementineAlqudah, Ahmad M.Lesser, Mark R.Alsadon, AbdullahLeszczuk, AgataAlsina, InaLeszczyńska, JoannaAltendorf, KaylaLetsiou, SophiaAlvarez Maldini, CarolinaLevi, AmnonAlvarez, AlejandraLewandowicz, GrazynaÁlvarez‐González, Carlos AlfonsoLexa, MatejÁlvarez-Mercado, Ana I.Leyva-Pérez, Maria de la OAlvarez-Suarez, JoseLi, ChaoAlves, ArturLi, GangAlves, SheilaLi, Man-WahAl-Yahyai, RashidLi, NingAlzubaidi, LaithLi, Po-HsienAmaral, Joana S.Li, Qian-ShengAmarowicz, RyszardLi, QiongyuAmato, MarianaLi, WeiAmbrožič-Dolinšek, JanaLi, XiangnanAmidžić Klarić, DanielaLi, YangAmirkhani, MasoumeLi, YangruiAmmar, MegahedLi, YanshengAmorós, AsunciónLi, ZhouAmpim, PeterLiagre, BertrandAmsbury, SamLiakopoulos, GeorgiosAmuji, Chinedu FelixLiao, Chien-SenAna, Fernández-OcañaLiao, Jyh-FeiAnbazhagan, SathiyaseelanLiao, Pei-ChunAncuceanu, RobertLiao, Wei-BiaoAnderson, AnneLiaudanskas, MindaugasAnderson, LarsLibantová, JanaAnderson, NeilLidon, FernandoAndongma, Awawing AnjwengwoLigor, MagdalenaAndrade Cetto, AdolfoLigrone, RobertoAndrade, GaldinoLim, JinheeAndreani, Nadia AndreaLim, Young SeokAndres, Maria FeLima, BeatrizAndrew, TedderLin, FanAngeletti, LorenzoLin, Hui-HsuanAngeli, SergioLin, Hung-YinAngelini, PaolaLin, Ming DerAngelini, RiccardoLin, Shian-RenAngelov, GeorgeLin, WeifengAngiolella, LetiziaLinacero, RosarioAnglani, FrancaLindberg, SylviaAnisimova, IrinaLingua, GuidoAnnibal, AndreaLinkevičius, EdgarasAntal, DianaLinkies, AdaAnthonymuthu, SelvarajLiovic, IvicaAntognoni, FabianaLipert, AnnaAntonino, José DijairLipowczan, MarcinAntonkiewicz, JacekLipsa, Florin DanielAntonova, DanielaLiu, AilinAntonucci, FrancescaLiu, BinbinAntunes, MauricioLiu, ChangAnver, ShajahanLiu, ChangningAnwar, AliLiu, FeiAoe, SeiichiroLiu, GuoquanApostolova, ElisavetaLiu, HongmeiApostolova, Emilia L.Liu, HuaAppels, RudiLiu, HuiAppendino, GiovanniLiu, LinshanAppezzato-da-Gloria, BeatrizLiu, NanaAraki, RyoichiLiu, SanzhenAranda, IsmaelLiu, ShaoyangArcalis, ElsaLiu, ShenkuiArcher, StephanieLlorens, EugenioAreche, CarlosLo Bianco, RiccardoArellano, Juan B.Lo Piccolo, ErmesArens, PaulLo Piero, Angela RobertaArianoutsou, MargaritaLo Schiavo, FiorellaAriel, FedericoLobiuc, AndreiArigò, AdrianaLobón, Natividad ChavesAriño, AgustínLocascio, AntonellaArkadiusz, StępieńLochmanová, GabrielaArkoun, MustaphaŁochyńska, MałgorzataArnason, JohnLohwasser, UlrikeArroyo, Antonio I.Loizzo, Monica R.Artyszak, ArkadiuszLojkova, LeaAsano, KazuhitoLokstein, HeikoAscrizzi, RobertaLombardo, LucaAsekova, SovetgulLombraña, Alba CuenaAshour, Mohamed L.Lone, Ajaz A.Ashraf, Muhammad ArslanLong, YuchenAshworth, Matt P.Lopes, Ana RitaAsíns, María JoséLopes, GracilianaAsnicar, FrancescoLopez, VictorAspée, Felipe JimenezLópez-Cepero, María RocaAtanassova, MariaLópez-Cristoffanini, CamiloAtanbori, JohnLopez-Girona, ElenaAta-Ul-Karim, Syed TahirLópez-Moya, Juan JoséAthanasios, DalakourasLoppi, StefanoAthar, Habib-ur-RehmanLora, JorgeAttila, FehérLord, EtienneAttila, HegedusLorenz, PeterAttorre, FabioLorenzis, Gabriella DeAugustine, Matthew P.Lorite, MariaAugustine, Sruthy MariaLoskutov, IgorAvanesyan, AlinaLotanna Micah, NnejiAversano, RiccardoLotina-Hennsen, BlasAvgerinos, KonstantinosŁotocka, BarbaraAvila-Cabadilla, Luis DanielLoveridge, JoelAvramidou, EvangeliaLožienė, KristinaAwasthi, PraveenLu, Pei-LuenAyala-Zavala, J. F.Lu, Wen-ChienAyyanath, Murali MohanLu, YouAzad, Obyedul KalamLucardi, RimaAzarin, Kirill VitalievichLuccini, LuigiAzevedo, HerlanderLucero, Leandro ExequielAzinheira, Helena G.Luci, GiacomoAziz, AzizLuciński, RobertAznar-Moreno, Jose AntonioLucioli, SimonaBaba, Abu ImranLuczaj, LukaszBabu, GiridharLudidi, NdikoBacelar, EuniceLudwiczuk, AgnieszkaBach, Thomas J.Luebert, FedericoBackhouse, DavidLuigi, MartaBácsi, IstvánLuis Jorge, Juan C.Baczewska-Dąbrowska, Aneta HelenaLukasiewicz, MarcinBadagliacca, GiuseppeŁukaszewska, AleksandraBader, AmmarLukaszewski, Adam J.Badiani, MaurizioLukatkin, Alexander S.Badjakov, IlianLukhovitskaya, NinaBadr, AbdelfattahLukić, IgorBae, Chang-HyuLukinac Čačić, JasminaBaeza, PilarŁukowski, AdrianBaghdadi, AliLumini, EricaBagniewska-Zadworna, AgnieszkaLuna, DiegoBahcevandziev, KirilLunerova, JanaBahuguna, AshutoshLuptakova, LenkaBailly, ChristianLurie, SusanBajaj, RuchikaLüthi, ChristophBajerová, PetraLüttschwager, DietmarBajguz, AndrzejLuzina, OlgaBakacsy, LászlóLyanguzova, IrinaBakoyiannis, IoannisŁyczko, JacekBalasubramani, Subramani ParanthamanLyu, Hong-KunBalasubramanian, BalamuralikrishnanLyu, Jae-IlBalažová, AndreaMa, DanBaldauf, Jutta A.Ma, FengyunBaldisserotto, AnnaMa, GuohuaBaldrich, PatriciaMa, GuojiaBalen, BiljanaMa, JunfengBalestrasse, KarinaMa, ZhaochengBalestrazzi, AlmaMaag, DanielBalestrini, Raffaella MariaMaalouf, Fouad S.Baležentienė, LigitaMacAlister, Cora A.Bálint, ErikaMachado, Michel MansurBallester, C.Machida-Hirano, RyokoBalusamy, Sri RenukadeviMacholdt, JannaBanach, ArturMacko-Podgórni, AlicjaBanchi, ElisaMacovei, AncaBandurska, HannaMacVean, Charles M.Bandyopadhyay, DebasishMadurapperuma, BuddhikaBanerjee, BikramMagerøy, MelissaBanilas, GeorgiosMaggini, RitaBankina, BirutaMagni, ChiaraBanko, PaulMago, RohitBankova, VassyaMagyar, ZoltanBanks, Jonathan M.Mahato, NeelimaBantis, FilipposMaheshwari, PritiBarabasz, WieslawMähler, NiklasBaragaño, DiegoMahmood, QaisarBaránek, MiroslavMaier, CameliaBaranova, EkaterinaMailer, RodBarantsevich, E. P.Maj, GrzegorzBarão, LúciaMajda, MateuszBarba, Francisco J.Majewska, MałgorzataBarba-González, RodrigoMajumdar, RajtilakBarbedo, Claudio JoséMakarenko, Maxim StanislavovichBarberini, SaraMakisha, NikolayBarbinta-Patrascu, M. E.Maksimov, Igor V.Barceló, Juan BarcelóMaksimović, VukBarcyte, DovileMakvandi, PooyanBareka, PepyMalacarne, GiuliaBari, EliaMalandrino, MeryBarinova, SophiaMalenica, NenadBar-Joseph, MosheMalfa, Giuseppe AntonioBarletta, EmanuelaMalik, SoniaBarłóg, PrzemysławMalinowski, RobertBarna, BalázsMalinowski, RyszardBarnes, PaulMalinská, Hana AuerBarone, EttoreMałkowski, EugeniuszBarr, Kelli L.Maloberti, AlessandroBarreca, DavideMandal, Mihir KumarBarreca, SalvatoreMandalari, GiuseppinaBarriocanal, CarlesMandolino, GiuseppeBarták, VojtěchMandrioli, RobertoBartha, SándorManfredini, StefanoBartish, Igor V.Mang, Stefania MirelaBartlow, Andrew W.Mangini, GiacomoBartnik, MagdalenaManivannan, AbinayaBartosova, LenkaMann, Rajinder S.Bartosz, GrzegorzMannino, GiuseppeBasal, OqbaMano, ShojiBashan, Luz E.Mano, YoshiroBashir, Muhammad AjmalManrique, EstebanBashir, SaqibMansourian, SuzanBaskin, Carol C.Mantzoukas, SpiridonBaslam, MarouaneManzaneda, Antonio J.Basnet, Bhoja RajMaoka, TakashiBassil, NahlaMarabottini, RositaBasso, DanielaMarasek-Ciolakowska, AgnieszkaBasu, DebaratiMarć, Małgorzata AnnaBasu, SaumikMarc, Romina AlinaBasu, SupratimMarchand, Patrice A.Batorska, MartynaMarchesini, Luca BelelliBattisti, RafaelMarchetti, MartaBatuman, OzgurMarchetti, NicolaBaturo-Ciesniewska, AnnaMarciniak, KatarzynaBatyk, IwonaMarcinkeviciene, AusraBaum, ChristelMarco, FranciscoBaum, MichaelMarczak, Barbara KrochmalBautista, InmaculadaMarczak, MałgorzataBazihizina, NadiaMarengo, AriannaBazos, IoannisMaresi, GiorgioBazylko, AgnieszkaMargaoan, RodicaBeamish, AlisonMargiotta, BenedettaBeaugrand, JohnnyMariam GaidBeaver, James S.Marian, EleonoraBeccari, GiovanniMarian, MalekBeck, AndreasMariani, LuigiBečka, DavidMarietta, FodorBedell, Jean PhilippeMarina, Anabel IsabelBegović, LidijaMarinaș, IoanaBehera, SmrutisanjitaMarinello, FrancescoBehnke-Borowczyk, JolantaMarino, GiadaBehr, MarcMarino, GiovanniBeier, Marcel PascalMarino, MarioBeilby, MaryMaris, LaubertsBeker, CezaryMarius, NiculauaBela, KrisztinaMariychuk, RuslanBeleggia, RominaMarkakis, EmmanouilBelimov, AndreyMarkou, GiorgosBellaloui, NacerMarkovic, DimitrijeBellot, María José GómezMarmiroli, MartaBeltramo, ChiaraMaroušek, JosefBelykh, Elena S.Marques, Ana CoelhoBelzile, FrançoisMarques, BrunoBen-Ari, GioraMarques, IsabelBen-Arie, RuthMarques, NatáliaBencivenga, StefanoMarquez-Rocha, Facundo J.Ben-Dor, ShifraMarrelli, MariangelaBenech-Arnold, Roberto LuisMarshall, Jordan M.Benedek, CsillaMarsico, Antonio DomenicoBenelli, CarlaMárta, LadányiBeni, ClaudioMartău, Adrian GheorgheBenítez, María JoséMartelli, GiuseppeBeola, LilianneMarti, LuciaBercu, MioaraMartín Matas, GuiomarBergero, RobertaMartin, Sara L.Berhow, MarkMartina, MatteoBeris, DespoinaMartínez Nicolas, Juan JoséBerjano, ReginaMartínez Rivas, Félix JuanBerlanga, Ángel MéridaMartinez Ruiz, CarolinaBernacchia, GiovanniMartinez, JuanBernard, Ernest C.Martínez-Force, EnriqueBernards, MarkMartínez-Hernández, FabiánBernstein, NiritMartínez‐Téllez, Miguel ÁngelBerova, MalgorzataMartín-Gil, JesúsBerrazaga, InsafMartins, IsabelBerretta, AndresaMartins, Maria Luisa LouroBertaccini, AssuntaMartins-Loução, Maria AméliaBertamini, MassimoMartz, FrancoiseBertea, CinziaMarzachí, CristinaBertero, DanielMarzec-Schmidt, KatarzynaBertin, SabrinaMas, Airy GrasBertini, LauraMasci, Valentina LaghezzaBerto, SilviaMasciocchi, NorbertoBertsouklis, KonstantinosMaserti, Bianca ElenaBęś, AgnieszkaMasi, MarcoBešlo, DragoMasias, Felipe BarriosBesnard, GuillaumeMaslin, BruceBessho-Uehara, KanakoMaslov, MikhailBetekhtin, AlexanderMasoud, AlaaBetti, MarcoMassa, BrunoBettoni, Jean CarlosMassimino Cocuzza, Giuseppe ErosBezerra, JadsonMassó, SergiBhaskara, Govinal BadigerMasuda, TatsuruBhati, Kaushal KumarMasuya, HayatoBhatia, DharmendraMata, RachelBhatt, PankajMatamoros, Manuel A.Bhattacharya, JhilikMateo-Bonmatí, EduardoBhattarai, KrishnaMatha, Shivaraj SheelavantaBhusal, NarayanMáthé, CsabaBi, GuihongMatilla, Angel J.Biagi, MarcoMatin, AnaBialik, RobertMatkowski, AdamBianchi, GiuliaMatny, OadiBianco, ArmandodorianoMatos, ManuelBianco, CarmelinaMatoša Kočar, MajaBiancolillo, AlessandraMatraszek-Gawron, RenataBiczak, RobertMatsui, HidenoriBiddick, MatthewMatsuoka, DaisukeBidel, LucMatthysse, Ann G.Biedermann, DavidMattias, GaglioBielecka, MonikaMattii, Giovan BattistaBielski, StanisławMattos, DirceuBiernasiuk, AnnaMatveeva, TatianaBiesiada, AnitaMatwijczuk, ArkadiuszBilia, Anna RitaMatyka, MariuszBilous, AndriiMaulana, FrankBilsborrow, PaulMavrogonatou, EleniBinelli, GiorgioMaxim, AurelBirarda, GiovanniMay, JaredBird, David Mck.Mayer, Juliana Lischka SampaioBiricolti, StefanoMazarji, MahmoudBirk, SebastianMazumder, KishorBirkeland, SiriMazzafera, PauloBirsa, Mihail LucianMcCabe, PaulBiscarini, FilippoMcCallum, JasonBiselli, ChiaraMcClaugherty, CharlesBisio, AngelaMcdonald, Glenn K.Bitonti, MariaMcKenzie-Gopsill, AndrewBitz, LidijaMcKirdy, SimonBizimungu, BenoîtMcletchie, NicholasBizzo, HumbertoMechchate, HamzaBjörn, Lars-OlofMechora, ŠpelaBlack, DavidMedeiros, Juliana S.Blaga, Alexandra CristinaMediavilla, IreneBlagodatski, ArtemMedina Juarez, Luis AngelBlagojevic, PolinaMedina, CarlosBlanco, AntonioMedina-Puche, LauraBlanco-Salas, JoséMedo, JurajBlando, FedericaMeena, Ram SwaroopBlanvillain, RobertMega, RyosukeBlindow, IrmgardMehraj, HasanBlinov, AlexandrMehta, AngelaBlinstrubienė, AušraMei, Kuo-ChingBlomme, JonasMeimandi, Maryam MozafarianBobis, OtiliaMeitzel, TobiasBobulska, LenkaMekhemar, MohamedBoccaccini, AlessandraMekinić, Ivana GeneralićBoccia, Antonella CaterinaMeletiou-Christou, Maria-SoniaBochnak, JustynaMelosik, IwonaBocianowski, JanMenale, BrunoBoczkowska, MajaMendes, Marta A.Bodale, IlieMendonça, DinaBode, RobertMenéndez, JulioBoden, ScottMeneses, CarlosBoer, Jennifer C.Menezes, Irwin Rose AlencarBogacz, AnnaMeng, XiaoxiBogdanov, Ivan V.Menghini, LuigiBoisivon, Helene RobertMenke, Aswin L.Bojić, MirzaMerah, OthmaneBojinov, BojinMercati, FrancescoBolarić, SnježanaMercuri, Anna MaríaBölcskei, HedvigMerecz-Sadowska, AnnaBoldizsár, ImreMerino, BeatrizBoldura, Oana-MariaMerry, RyanBonciu, ElenaMesbah, MortezaBonello, FedericaMešić, AleksandarBonvicini, FrancescaMessyasz, BeataBorak, BeataMészáros, KláraBorek, SławomirMevy, Jean-PhilippeBorger, CatherineMeyer, Roland D.Bormashenko, EdwardMezzetti, BrunoBoroń, DariuszMiao, YuBorowska-Wykręt, DorotaMiazga-Karska, MalgorzataBorrelli, Grazia MariaMiceli, AlessandroBorsai, OrsolyaMiceli, AntonioBortolami, MartinaMichail, MichailidisBörve, JorunnMichailidis, MichailBoscaiu, MonicaMichalak, MarcinBosi, GiovannaMichel, PiotrBotez, ElisabetaMicheletti, DiegoBottini, RubénMicillo, RaffaellaBotto, AnnaMielniczuk, ElżbietaBouaïcha, NoureddineMieog, Jos CorneliusBoualem, AdnaneMierek-Adamska, AgnieszkaBougrin, KhalidMigliore, JérémyBouranis, Dimitris L.Migliore, LucianaBouzouidja, RyadMigliori, Carmela AnnaBowden, RichMiguel, Maria Da Graça CostaBowman, DarylMigunova, Varvara D.Bozena, SeraMihai-Leonard, DudumanBozin, BiljanaMihailova, GerganaBrader, GünterMihál, IvanBraglia, LucaMihali, CristinaBrambilla, FabioMihaylova, DashaBranca, FerdinandoMikhail, GenaevBrandão, Maria Das Gracas LinsMikhailova, ElenaBranowska, DanutaMikołajczak, KrzysztofBravo, Susana B.Mikołajczyk-Bator, KatarzynaBrazaitytė, AušraMikšová, RomanaBreia, RichardMilani, AndreaBreiterová, KateřinaMilella, LuigiBresta, PanagiotaMiler, NataliaBrestic, MarianMileva, MilkaBrestič, MariánMilioni, DimitraBretfeld, MarioMilito, AlfonsinaBrieba, LuisMillan Almaraz, Jesus RobertoBriedé, JaccoMiller, GloriaBrígido, ClarisseMilosavljević, IvanBrito, CátiaMinafra, AngelantonioBritvec, MihaelaMinamikawa, MaiBrkljača, MiaMinato, Ken-ichiroBroberg, MartinMinelli, AlessandroBrophy, Joseph JohnMinicka, JuliaBrouard, IgnacioMinkina, TatianaBrown Miyara, SigalMino, MasanobuBrown, Shawn P.Minto, Robert E.Brownlee, Iain A.Miralles, PabloBrück, HolgerMiranda, AnaBruez, EmilieMiranda-Apodaca, JonBrullo, SalvatoreMircea, CorneliaBrumos, JavierMirmazloum, ImanBrundu, GiuseppeMiroshnichenko, Dmitry N.Brunetti, CeciliaMisaki, RyoBrunner, SusanneMišan, AleksandraBruno, MaurizioMishra, Amit KumarBryła, MarcinMishra, AwdheshBuades Rubio, Jose MariaMishra, DivyaBubici, GiovanniMishra, PriyankaBubola, MarijanMishra, PuneetBucar, FranzMishra, SasmitaBucchini, Anahi Elena AdaMisra, Marendra NarayanBuck, WilliamMisra, Rajesh ChandraBuczek, JanMitakou, SofiaBudahn, HolgerMitić, BoženaBudak, HikmetMitliagka, ParaskeviBudeanu, MariusMitrea, LauraBūdienė, JurgaMitrović, MiroslavaBudzynska, SylwiaMitsuyama, SusumuBueno, MilagrosMiura, ChihiroBueno-Silva, BrunoMiyanaga, AkimasaBuesa Pueyo, IgnacioMiyazawa, ShinichiBuijs, GondaMizzotti, ChiaraBujdoso, GezaMleczek, MiroslawBulak, PiotrMlinarić, SelmaBuldrini, FabrizioMoccia, StefaniaBulychev, Alexander A.Modak, BrendaBunce, James A.Mohamed, HebaBunea, AndreaMohanraj, RemyaBurachevskaya, MarinaMohanty, BijayalaxmiBurcul, FrankoMoisa, CristianBurczyk, JarosławMoitzi, GerhardBurducea, MarianMok, Daniel Kam-WahBurgess, Alexandra JacquelynMoldovan, BiancaBurgos, LorenzoMolina Salinas, Gloria MaríaBurlakovs, JurisMolina, LázaroBurrows, Geoffrey E.Molina-Heredia, Fernando P.Bursać Kovačević, DanijelaMolinier, JeanBursic, VojislavaMoliszewska, EwaBurt, ChrisMomčilov, Milana TrifunovićBusoms, SilviaMónaco, CeciliaBustamante, Ramiro O.Monasterio, Romina PaulaButnariu, MonicaMonokrousos, NikolaosByun, ChaehoMonreal, José A.Cabaleiro, ManuelMontalbán, Itziar A.Cacak-Pietrzak, GrażynaMontana, Angel M.Cacciola, FrancescoMontemurro, CinziaCadenas Pliego, GregorioMontes, NuriaCagáň, ĽudovítMontesinos, Juan CarlosCagliero, Cecilia LuciaMonteuuis, OlivierCahlíková, LucieMontiel, GrégoryCai, GiampieroMoon, SunokCai, HongmeiMoore, JohnCai, LimingMoore, MartenCal, MagdalenaMora Garcia, SantiagoCalatayud, AngelesMora, DemetrioCalderini, OrnellaMora, FreddyCalderon, RocioMorais, João Paulo SaraivaCaldo, Kristian MarkMorais, Maria CristinaCalero, SaraMoral, JuanCalevo, JacopoMorales, LauraCalha, Isabel M.Morales-González, José AntonioCalvo-Polanco, MonicaMora-Montes, HectorCalzarano, FrancescoMorán-Diez, María E.Čamagajevac, Ivna ŠtolfaMoraru, Paula IoanaCamargo Rodríguez, Anyela ValentinaMoravcikova, JanaCamele, IppolitoMoreno Saiz, Juan CarlosCampa, ClaudineMoreno, AdrianCampanella, JamesMoreno, DanielCampbell, MichaelMoreno-Rojas, José ManuelCampbell, ShaneMori, KazuhiroCampilho, AnaMori, MauroCampillo-Cora, ClaudiaMoricz, AgnesCampion, BrunoMorita, KyojiCampisi, PatriziaMorita, ShigetoCampos, DeboraMorkunas, IwonaCampos-Vargas, Reinaldo Campos-VargasMorohashi, KengoCanales, JavierMorreale, GiacomoCanhoto, JorgeMoschou, Panagiotis NikolaouCanini, AntonellaMoser, ClaudioCannon, RichardMossel, EricCano Lamadrid, MarinaMostowska, AgnieszkaCantamessa, SimoneMot, AugustinCantini, ClaudioMota, Manuel M.Cantos, ManuelMotti, RiccardoCaparrotta, StefaniaMottola, FilomenaCapasso, RaffaeleMouga, TeresaCapece, AngelaMourato, Miguel P.Cappiello, MarioMourtzinos, IoannisCaprari, ClaudioMousivand, AliCapraro, FlavioMoustakas, MichaelCarabetta, SoniaMouzeyar, SaïdCarac, GetaMoyankova, DanielaCarata, ElisabettaMożdżeń, KatarzynaCarbas, BrunaMroczek, AgnieszkaCarbone, FabrizioMucalo, AnaCarbone, KatyaMuccifora, SimonettaCarbonell-Bejerano, PabloMucha, JoannaCárdenas Pérez, StefanyMugerwa, HabibuCardoni, MartinaMuhovski, YordanCardoso, AmandaMuktadir, Md AbdulCardoso, HéliaMulas, MaurizioCardoso, Jean CarlosMulet, Jose M.Cardoso, PauloMüller, CarstenCaretto, SofiaMuller, KarelCarev, IvanaMüller, Lena MariaCarimi, FrancescoMüller, Maren LilianCarletti, PaoloMuminjanov, HafizCarluccio, Anna VittoriaMuneer, SowbiyaCarmen, MejíaMunekata, Paulo E. S.Carnevale Neto, FaustoMuñoz, JesúsČarni, AndražMuñoz, KatherineCarranca, CorinaMupambi, GiversonCarriquí, MarcMurata, JunCarrubba, AlessandraMurata, KazuyaCarruggio, FrancescaMuravnik, Lyudmila E.Carter, BenjaminMuresan, CrinaCartmill, Andrew D.Muresan, Mariana EugeniaCarullo, GabrieleMurias, MarekCaruso, GiovanniMurray, BradCaruso, MarcoMurshid, NimerCaruso, PaolaMusić, Martina ŠerugaCarvalho, LuísaMussury, Rosilda MaraCarvalho, MárciaMustafa, AdnanCasado, NataliaMustroph, AngelikaCasanova, Michelle T.Muszyńska, EwaCasapullo, AgostinoMuthusamy, MuthusamyCasas, Ana M.Mutiga, Samuel K.Casas, José LuisMuto, AntonellaCasey, Philip S.Muzzio, NicolásCassol, DanielaMylona, PhotiniCastañeda, CarmenMylonas, IoannisCastellano, GloriaMyskow, BeataCastellanos, AlejandroNacheva, Lilyana R.Castilho, PaulaNacry, PhilippeCastillo, AnaNadeem, Muhammad AzharCastillo, AraceliNafis, AhmedCastillo, PabloNagahage, IsuraCatalano, CaterinaNagamitsu, TeruyoshiCatarino, Marcelo D.Nagatsu, AkitoCatry, FilipeNagel, RaimundCattani, Douglas J.Nagl, NevenaCavaleiro, CarlosNagy, IstvanCavalieri, AndreaNakaba, SatoshiCazacu, AnaNakahara, Kenji S.Cazetta, Jairo O.Nakamura, Shin-ichiCebola, Maria JoãoNakano, MichiharuCecchi, GraziaNakashima, SouichiCecchini, NicolasNakatsukasa, KunioCeci, AndreaNanasato, YoshihikoĆelepirović, NevenkaNankar, AmolCellini, AntonioNanos, George D.Čepková, Petra HlásnáNapierala, MartaČepulienė, RitaNapierala, MichalCerenius, LageNapoli, MarcoČerevková, AndreaNardi, SerenellaČerný, MartinNardini, AndreaCerrato, AndreaNardozza, SimonaCerri, MartinaNarmani, AbolfazlCervantes, EmilioNarouei-Khandan, Hossein A.Cesarino, IgorNash, RobertCesbron, SophieNashar, MilkaČesonienė, LaimaNasreddine, RoubaCéspedes Acuña, Carlos LeonardoNatarajan, PurushothamanCessna, StephenNausch, HenrikCetera, PaolaNavarro, EstanisChaban, InnaNavarro-León, EloyChabbert, BrigitteNavrátil, JosefChaki, MouniraNawade, BhagwatChakrabarti, SubhadeepNawaz, Muhammad AmjadChamrád, IvoNawirska-Olszańska, AgnieszkaChandnani, RahulNawrocka, JustynaChandra Roy, NepalNawrot-Chorabik, KatarzynaChandrasekaran, MurugesanN'Diaye, AmidouChandrasekaran, Srinivas NiranjNebija, DashnorChang, ChengŅečajeva, JevgenijaChang, Chia-cheNecas, TomasChang, Ching-chunNechita, ConstantinChang, Ching-yaoNegri, GiuseppinaChang, Hao-XunNegro, CarmineChang, Ing-FengNeilson, RoyChardon, FabienNeiva, Duarte M.Charles Richard, John LalithNellist, CharlotteChatzidimopoulos, MichaelNelson, MatthewChatzistathis, TheocharisNemes, DánielChaurasia, SavitaNemeskéri, EszterChaves, BernardoNémeth, András GyörgyChávez-Servia, José LuisNerva, LucaChazot, PaulNeto, CarlosChedea, Veronica SandaNewman, Steven E.Cheimona, NikolinaNgo, Hien T. T.Chekanov, KonstantinNiazian, MohsenChen, Bang-YuanNicoletti, MarcelloChen, Chang-ChangNiculae, MihaelaChen, Chien-TehNiebel, AndreasChen, Fure-ChyiNiedbała, GniewkoChen, GengjunNiedojadło, JanuszChen, GuangshuiNiedzielski, PrzemysławChen, Hsin-ChunNiemann, JanettaChen, Jen-TsungNiemczak, MichalChen, Liang-YuNiewiadomska, EwaChen, PeichenNigro, FrancoChen, Peng-WenNikolic, LjubisaChen, Ruey ShyangNikolić, NebojšaChen, TongNikoloudakis, NikolaosChen, Tsan-ChiNimmakayala, PadmaChénais, BenoitNin, StefaniaCheng, AcgaNina, BrutchCheng, Juei-TangNinou, ElissavetCheng, Yuan-BinNishi, KosukeChertov, OlegNishii, KanaeChetverikov, SergeyNishizawa-Yokoi, AyakoChhapekar, SushilNistor, CristinaChianese, GiuseppinaNistor, EleonoraChiarini, LuigiNistor, Oana ViorelaChiba, YukihiroNita, MizuhoChibbar, RavindraNitulescu, GeorgianaChica, Rosa MaríaNiu, FurongChicca, MilviaNobre, Marcos Augusto LimaChien, Mei-FangNocito, Fabio F.Chilosi, GabrieleNoda, MasafumiChim, Bee KhimNoda, TakahiroChin, Joong HyounNogales-Bueno, JulioChiou, Chun-TangNoman, AliChira, NicoletaNoman, MuhammadChirico, Giovanni BattistaNoor, Mehmood AliChiumenti, MichelaNorton, Sally L.Chmara, RafałNosov, AlexanderChmelikova, L.Noszczyńska, MagdalenaChmielowska-Bąk, JagnaNovák, JanChmura, DamianNovello, GiorgiaCho, Myeong-CheoulNovikov, AndriyCho, NamkiNovikov, ArthurChofong, Gilbert NchongbohNovikova, Lubov YuChoi, DongsuNovo, LuísChoi, Ik-youngNovoselovic, DarioChoi, Il-RyongNowacka, MalgorzataChoi, Yong-KeunNowacka-Jechalke, NataliaChoob, Vladimir V.Nowak, AnnaChorianopoulou, Styliani N.Nowak, ArkadiuszChoudhary, ShrutiNowak, MichałChoudhury, Swarup RoyNowicka, AnnaChowaniak, MaciejNowosad, KamilaChris, CockelNtinas, GeorgiosChristoffers, MichaelNuc, KatarzynaChristopher, MandyNuc, PrzemysławChristos, ChatzisavidisNunes, Leonel Jorge RibeiroChudzińska, EwaNunes, Maria LeonorChung, GyuhwaNunes, MartaChung, HsiaohangNunziata, AngelinaChung, KwiMiNurzyńska-Wierdak, RenataChwil, MirosławaNyine, MosesChyau, Charng-CherngOancea, FlorinCiancio, AurelioObojes, NikolausCianfaglione, KevinOborník, MiroslavCiarmiello, LoredanaO'Brien, ChrisCiccoritti, RobertoOcchialini, AlessandroÇiçek, Serhat SezaiOchatt, SergioCichowska, JoannaOchoa-Alejo, NeftalíCiereszko, IwonaOddo, ElisabettaCillo, FabrizioOdonne, GuillaumeCimpoiu, ClaudiaOelmuller, RalfCioanca, OanaOgata, YoshiyukiCipakova, IngridOgiso-Tanaka, EriCirebea, MirelaOgórek, RafałCires, EduardoOgrodowicz, PiotrCiulla, MicheleOguchi, RiichiCladis, DennisO'Hara, AndrewClassen, BirgitOhkama-Ohtsu, NaokoClassen, John J.Ohmido, NobukoCleal, ChristopherOhtomo, RyoClemente, MarinaOjeda Alayon, Dario I.Clericuzio, MarcoOkada, TaketoCliment, JoséOkazawa, AtsushiCliquet, Jean-BernardOkińczyc, PiotrClissold, Fiona J.Oklešťková, JanaClode, PetaOkoń, SylwiaCocan, IleanaOkorski, AdamCocucci, MaurizioOkunade, Adewole L.Codina, GeorgianaOlaetxea, MaiteCoelho, NatachaOláh, RóbertCoelho, Paula S.Oláh, ViktorCohan, Jean-PierreOlaru, Octavian TudorelCohen, Ana CarmenOlczyk, PawełCohen, James I.Olech, MartaCointault, FrédéricOlejniczak, IzabellaCole, Logan W.Oleksa, AndrzejColecchia, Salvatore AntonioOlennikov, DaniilColinas, CarlosOlivares, Barlin O.Collani, SilvioOliveira, CristinaCollin, BlancheOliveira, PauloCollinge, David B.Oliveira, Rui S.Comerford, NicholasOlszewska, Monika A.Cominelli, EleonoraOng, Eng ShiCommichau, FabianOniszczuk, AnnaConcepcion, Jeanaflor CrystalOnodera, TakashiConesa, María R.Onwude, Daniel I.Confalonieri, MassimoOnwudiwe, DamianConklin, Patricia L.Oprică, LăcrămioaraConner, JoannOracz, JoannaConnolly, AlisonOramas-Royo, SandraConran, JohnOrdas, BernardoConstantinidis, TheophanisOrdaz-Ortiz, José JuanContaldo, NicolettaÖrdög, AttilaConti, StefanoÖrdög, VinceContreras, Rodrigo A.O’Rear, EdgarCopetta, AndreaOren, EladCopolovici, LucianOrfanidou, ChrysoulaCör, DarijaOrłowska, RenataCordea, MirelaOroian, Mircea AdrianCordeiro, MarcosOrtega, Sara FrancoCornea-Cipcigan, MihaielaOsabe, KenjiCornejo, PabloOsei-Bonsu, IsaacCornelius, Mary L.Oskay, Rıza GörkemCorrêa, Camila RenataOssowicki, AdamCorredoira, ElenaOstaszewska-Bugajska, MonikaCorreia, Carlos M.Ostrowska-Ligȩza, EwaCorreia, Pedro JoséOtaka, JunnosukeCortis, PierluigiOtulak-Kozieł, KatarzynaCossignani, LinaOuvrard, PierreCosta, JoséOuyabe, MichelCosta, Maria Do CéuOvidi, ElisaCosta, Rita LourencoOyama, Kin-ichiCostinel, DianaOzarowski, MarcinCozzolino, EugenioOzcelik, MineCravero, Maria CarlaPacak, AndrzejCreamer, RebeccaPace, LorettaCrespi, MartinPacheco, RitaCrespo, Manuel BenitoPacifico, DavideCristiano, GiuseppePaciolla, CostantinoCroce, Anna CletaPacuta, VladimirCrupi, PasqualePaczos-Grzeda, EdytaCruz, RodrigoPadilla, Isabel Maria GonzalezCruz-Chamorro, IvanPadilla-Gonzalez, GuillermoCruz-Ramirez, AlfredoPagán, IsraelCseko, KataPagano, LucaCséplő, ÁgnesPagano, MarioCseresnyés, ImrePageau, KarineCsiszár, JolánPais, Maria SaloméCucina, MirkoPaiva, Élder A. S.Cucinotta, MaraPajoro, AliceCuena-Lombraña, AlbaPál, MagdaCuesta, CandelaPalacio, SaraCuevas, JuliánPalčić, IgorCui, RongfengPalko-Labuz, AnnaCunha, JorgePalkovics, LászlóCunniffe, NikPallag, AnnamáriaCurci, MaddalenaPalleria, CaterinaCurtis, LukePalma, LeopoldoCwalina-Ambroziak, BożenaPalomo-Ríos, ElenaĆwieląg-Piasecka, IrminaPampana, SilviaCzako, MihalyPan, ChangtianCzaplicki, SylwesterPanda, KaushikCzarniecka-Skubina, EwaPandey, DhananjayCzernicka, MałgorzataPandey, SadanandCzerniewicz, PawełPane, CatelloCzerwik-Marcinkowska, JoannaPanek, JacekCzerwińska, MonikaPanero, JoseCzortek, PatrykPanitsa, MariaCzubacka, AnnaPanja, SaumikDa Silva, Anabela BernardesPanno, StefanoDabrowski, PiotrPantelidis, GeorgiosDąbrowski, PiotrPanten, KerstinDadáková, KateřinaPanteris, EmmanuelDafni, AmotsPantha, PramodDagnon, SoleyaPaoli, LucaD'Agostino, NunzioPaolo, DarioDahlin, PaulPapadia, ParideDahoumane, SiPapathanasiou, FokionDai, YuminPapenbrock, JuttaDalCorso, GiovanniPapini, AlessioD'Alessandro, StefanoPaponov, Ivan A.Dallinger, ReinhardPapp, IstvánDama, MuraliPappas, ChristosDana, ChitimusParaschiv, MariusDang, JunParashuraman, SeetharamanDaniela, AliotoParedes-López, OctavioDanielski, RenanParis, RobertaDanova, KalinaPark, Chong-WookD'Aprile, FabrizioPark, InkyuDaras, GerasimosPark, Jin HeeDarenskaya, Marina AleksandrovnaPark, Jong-HwaDarko, Daniel A.Park, JongseokDarkó, ÉvaPark, Kyoung-ChanDarnowski, DouglasPark, Kyung-MokDarouich, HanaaPark, Sang WookDarras, AnastasiosPark, Sang-WookDas, Shaon KumarPark, Seon-JooDas, SubhaParra, AntonioDastogeer, KhondokerPartzsch, MonikaDatta, RahulParus, AnnaDavid, UriarteParveen, AmnaDavis, Aaron P.Parvin, KhurshedaDavolos, DomenicoPârvu, MarcelDavydenko, KaterynaParzymies, MarzenaDay, DavidPascual, LauraDe Alba, Ángel Emilio MartínezPascual‐Seva, NúriaDe Andrade, EugéniaPasechnik, TatyanaDe Bellis, LuigiPashkovskiy, PavelDe Biasi, Margherita G.Pasin, Fabiode Bittencourt Pasquali, Matheus AugustoPasković, IgorDe Bruno, AlessandraPasqua, GabriellaDe Camargo, Adriano CostaPassafiume, RobertaDe Caroli, MonicaPassalacqua, Nicodemo G.De Corato, UgoPassera, AlessandroDe Curtis, FilippoPatanè, CristinaDe Deurwaerder, HannesPatel, Jai SinghDe Freitas Fraga, Hugo PachecoPaternostro, FerdinandoDe La Torre, AmandaPaterson, AndrewDe La Varga, HerminiaPatil, Abhinandan SurgondaDe Lacerda, Claudivan FeitosaPatra, Jayanta KumarDe Luca, DanielePattnayak, Kanhu CharanDe Luca, FrancescaPatykowski, JacekDe Luca, PasqualePaule, LadislavDe Lucia, BarbaraPaulette, LauraDe Lurdes Inácio, MariaPauletti, GabrielDe Masi, LuigiPavela, RomanDe Mieri, MariaPaventi, GianlucaDe Oliveira Júnior, Rubem SilvérioPavlov, DanailDe Padova, PaolaPavlovič, AndrejDe Pádua Teixeira, SimonePaweł'kowicz, Magdalena E.De Paola, DomenicoPawlik, AnnaDe Paolis, AngeloPawlik-Skowrońka, BarbaraDe Rogatis, AnnaPawluśkiewicz, BogumiłaDe Santis, Michele AndreaPazzagli, LuigiaDe Sousa, ÂngelaPearce, NorrieDe Sousa, Damião PergentinoPearce, StephenDe Souza, Danival JoséPedatella, SilvanaDe Tommasi, NunziatinaPedersen, HenrikDe Tullio, Mario C.Pejić, IvanDe Vienne, DominiquePék, ZoltánDe Vries, JanPellegrini, MarikaDe Zwart, FeijePeña, AránzazuDeane, David C.Peňázová, EliškaDecroo, CorentinPeng, FoenDegl'innocenti, DonatellaPeng, JiangnanDegola, FrancescaPeralta, RosaneDeineko, Elena VictorovnaPereira, Ana MartaDel Buono, DanielePereira, JoseDel Giudice, RitaPereira, PaulaDel Moral Torres, FernandoPereira, Renato B.Délano-Frier, John PaulPereira, Susana S.Delgado Valerio, PatriciaPerelomov, Leonid V.Delhomme, NicolasPerestrelo, Rosa Maria De SáDeligios, Paola A.Pérez Artés, EncarnaciónDelis, CostasPérez De Castro, AnaDello Ioio, RaffaelePerez De Souza, LeonardoDell'olmo, ElianaPérez Reyes, CarolinaDelmas, FredericPérez-Bueno, MarisaDemmig-Adams, BarbaraPérez-Correa, José RicardoDenev, PetkoPérez-Fernández, MaríaDeng, XinPérez-García, Francisco J.Denisow, BozenaPérez-Marcos, MaríaDepeint, FlorePérez-Montaño, FranciscoDerewiaka, DorotaPérez-Romero, Luis FelipeDeshmukh, RupeshPerkin, Lindsey C.Desseva, IvelinaPerkins-Veazie, PenelopeDessureault-Rompré, JacynthePerlikowski, DawidDestandau, EmiliePernas, MonicaDever, JanePernisová, MarkétaDevkota, Hari PrasadPerreau, FrançoisDevlamynck, ReindertPerrella, GiorgioDevlin, PaulPerrino, EnricoDhariwal, RamanPerrone, GiancarloDhungana, SanjeevPerry, SharynDi Gaetano, SoniaPers-Kamczyc, EmiliaDi Giacomo, ClaudiaPerucka, IrenaDi Gioia, FrancescoPessoa, Maria FernandaDi Gregorio, SimonaPetar, PujićDi Lecce, GiuseppePetrellis, NikosDi Mambro, RiccardoPetronilho, SílviaDi Maro, AntimoPetropoulos, SpyridonDi Martino, CatelloPetrova, SlaveyaDi Marzo, MaurizioPetruczynik, AnnaDi Mola, IdaPetruzzelli, GianniantonioDi Nunzio, MattiaPetruzzellis, FrancescoDi Piazza, SimonePetry, MirtaDi Rita, FedericoPetti, CarloalbertoDi Salle, AnnaPezzarossa, BeatriceDi Sanzo, RosaPfeifer, LukasDi Venere, DonatoPham, Anh TungDiamond, GillPhan, Huyen T. T.Dias, Cindy Leonor AlvesPhillips, DylanDias, Elisabete FurtadoPiasecki, CristianoDias, Fernanda F. G.Piątczak, EwelinaDias, Maria CelestePicchio, RodolfoDíaz Esteban, MarioPiccinelli, Anna LisaDíaz García, PamelaPiccolella, SimonaDiaz, EsperanzaPichler, AnitaDiaz, GiselaPicq, SandrineDiaz-Garcia, LuisPiechalak, AnetaDiaz-Lara, AlfredoPiecuch, AgataDiaz-Vivancos, PedroPiedras, PedroDib, Julián RafaelPielech, RemigiuszDibwe, Dya FitaPiepho, Hans-PeterDiederichsen, AxelPietr, StanisławDifonzo, GrazianaPietras, MarcinDijk, AaltPietrowska-Borek, MałgorzataDillman, Adler R.Pignata, GiuseppeDinca, LucianPilkington, LisaDincheva, IvaylaPillai, SreejaraniDinis, Lia Tânia JPilu, RobertoDireito, RosaPiluzza, GiannellaDitta, AllahPiñero-Dalmau, DanielDivashuk, MikhailPinheiro, JoaquinaDixit, NaveenPini, ElenaDługosz, MarekPinker, InaDługosz-Grochowska, OlgaPiński, ArturDobosz, RenataPintea, AdelaDobrikova, AneliaPinto Cruz, CarlaDobrowolski, Jan W.Pinto Gomes, CarlosDobryszycki, PiotrPinto, Diana CláudiaDobson, Heidi E. M.Pinto, EdgarDocea, AncaPinto, LorisDodd, Richard S.Pinto, LuísDolatabadian, AriaPintus, FrancescaDoležal, KarelPinzaru, IuliaDołowy, MałgorzataPiotrowicz-Cieślak, AgnieszkaDomenici, GiacomoPipan, BarbaraDomina, GianniantonioPiro, AmaliaDomingues, Douglas SilvaPirona, RaulDominika, BednarczykPistelli, LauraDomonkos, IldikóPitura, KarolinaDonati, IrenePizo, Marco AurélioDong, HongxuPlata-Rueda, AngelicaDong, L. (Lemeng)PŁażek, AgnieszkaDordevic, DaniPlíhal, OndřejDos Santos Lima, MarcosPłotka-Wasylka, Justynados Santos Nascimento, Luana BeatrizPobiega, KatarzynaDos Santos, Maria Clara VieiraPoblaciones, MariaDosoky, NouraPoblete-Grant, PatriciaDouda, OndřejPociecha, EwaDougherty, LauraPodar, DorinaDougnon, GodfriedPodkowiński, JanDouma, MountasserPodleśny, JanuszDrabińska, NataliaPodolska, GrażynaDrava, GiulianaPodwyszynska, MalgorzataDresler, SławomirPogány, MiklósDrochioiu, GabiPogorzelec, MagdalenaDrzewiecka, KingaPogrzeba, MartaDuan, JialeiPohlmeier, AndreasDuarte, Maria CristinaPoiana, Mariana-AtenaDubeaux, GuillaumePoilane, ChristopheDubey, AnamikaPokhrel, LokDubois, AnnickPokovai, KlaraDubrovina, AlexandraPolák, BeataDuchêne, EricPoljak, IgorDuchowicz, PabloPolkowska-Kowalczyk, LidiaDucsay, LadislavPolle, Jurgen E. W.Duda, Marcel MateiPolyakov, NikolayDudas, SlavicaPonder, AlicjaDulf, FranciscPonert, JanDuliu, OctavianPonnu, JathishDumancas, GerardPonte, NunoDumičić, GvozdenPoór, PéterDumitriu, Georgiana-DianaPoosapati, SowmyaDunić, Jasenka AntunovićPop, Lecturer Carmen RodicaDunkić, ValerijaPopa, Dana ElenaDuralija, BorisPopek, RobertDurante, MirianaPopescu, Paul-AlexandruDurdevic, BorisPopłońska, KatarzynaDurgo, KsenijaPopova, AnetaDurian, GuidoPopova, Elena V.Đurović, SašaPopović, MarijanaD'Urso, GildaPorceddu, MarcoDuso, CarloPoretsky, EllyDutu, LigiaPorfírio, SaraDvojković, KrešimirPorro, DuilioDwiyanti, Maria StefaniePortillo López, AmeliaDziadas, Mariusz TomaszPortugal, AntonioDziedzic, KrzysztofPoschenrieder, CharlotteEamens, Andrew L.Potaniec, BartłomiejEbeed, HebaPotenza, GiovannaEbert, AndreasPotocký, MartinEbihara, AtsushiPotokina, ElenaEchegaray, Erik RPott, Delphine M.Eckerstorfer, MichaelPotter, DanielEderli, LuisaPoulios, StylianosEdmé, Serge J.Poussereau, NathalieEdwards, StellaPower, SimonEffah, EvansPozzi, CarloEftimov, MiroslavPramanik, DibyajyotiEgamberdieva, DilfuzaPratley, JimEgerton-Warburton, LouisePraus, LuděkEgipto, RicardoPreiner, DarkoEilers, Elisabeth JohannaPreiti, GiovanniEiras, MarceloPrerostova, Mgr. SylvaEisenback, Jonathan D.Priatama, RyzaEisenhaber, FrankPrieto, Jose M.Eitle, Markus W.Prieto, MiguelEklund, Patrik ChristofferPrieto, Miguel ÁngelEl Chami, DanielPrigigallo, Maria IsabellaEl Khalloufi, FatimaPrimi, RiccardoEl Obeid, DanyPrisa, DomenicoEl-Aarag, Bishoy Y. A.Prkić, AnteEl-Ansary, HosamProbanza, AgustinElansary, Hosam O.Procházková, LenkaEl-Esawi, Mohamed A.Prockow, JaroslawElhady, AhmedProestos, CharalamposEliades, MarinosProietti, SimonaEliášová, KateřinaProsekov, AlexandrEliopoulos, Panagiotis A.Prudent, MarionElKelish, AmrPrusky, DovElkington, BethanyPrzemieniecki, SebastianElkins, KellyPrzemysław, NucEller, FranziskaPrzybył, JarosławEllis, Thomas Henry NoelPrzygodziński, PrzemysławEl-Mochtar, ChoaaPtushenko, Vasilii VitalievichEl-Ramady, HassanPuangmalai, NichaElsayed, MansourPucci, NicolettaEl-Seedi, Hesham R.Puccinelli, MartinaElshamy, AbdelsamedPuchalka, RadoslawEl-Sharkawy, IslamPucker, BoasElsharkawy, MohsenPuente Luna, IvánElvira, SusanaPuglia, GiuseppeElzaawely, Abdelnaser A.Puig, Carolina G.Emeriewen, Ofere FrancisPuigdomènech, PereEndresen, DagPunia, SnehEngelberth, JurgenPuntieri, JavierEnomoto, GenPuri, AkshitEnrique, Martinez-ForcePurushothama, Charith Raj AdkarEra, BenedettaPyka, AlinaErcisli, SezalQaderi, MirwaisErickson, David L.Qayyum, Muhammad AbdulErinle, KehindeQazi, SohailErland, LaurenQian, HongErsilia, AlexaQiao, PengfeiErzsébet, DomokosQin, YonghuaEshel, AmramQiu, MinghuaEskandari, SamiehQu, CunminEsmat, AhmedQuadrana, LeandroEspada-Bellido, EstrellaQualset, Calvin O.Espen, LucaQuan, Nguyen VanEsperon-Rodriguez, ManuelQuéro, AnthonyEspinosa-García, Francisco JavierQuerol Audí, JordiEs-Safi, Nour EddineQuijada-Garrido, IsabelEsteve Ràfols, MontserratQuinet, MurielEsteves, Ana CristinaQuiñones, Miguel A.Estrada Camarena, ErikaQuintero Salazar, BacilizaEsumi, TomoyaQuiquampoix, HervéEtienne, PhilippeQuiroga, GabrielaEulenstein, FrankRaal, AinÉva, CsabaRaccuia, Salvatore AntoninoEvangelisti, EdouardRace, MarcoEvans, Barbara R.Racuciu, MihaelaEzra, DavidRadan, MilaEzzati, SattarRadócz, LászlóFabiani, RobertoRadosavljević, IvanFabroni, SimonaRadu, NicoletaFail, JózsefRadulov, IsidoraFailla, OsvaldoRady, MostafaFalcão, Soraia I.Rafique, JamalFalistocco, EgiziaRago, DanielaFalsini, SaraRagonezi, CarlaFan, LushengRagusa, AndreaFanfarillo, EmanueleRagusa, SalvatoreFang, DavidRaguso, RobertFanizzi, Francesco PaoloRahman, HabibFanourakis, DimitriosRahman, Inayat UrFaraone, ImmacolataRahman, Mohammad AtikurFaraoni, PaolaRahman, Muhammad Habib UrFarcas, Anca CorinaRai, KrishanFarias-Pereira, RenalisonRai, RikkyFarkaš, VladimírRaimondo, Maria LuisaFarooq, ShahidRaj, DanutaFarzaneh, VahidRajarapu, Swapna PriyaFascella, GiancarloRajendra Kopalli, SpandanaFascetti, SimonettaRajesh, YarraFateryga, AlexanderRajoka, Muhammad Shahid RiazFatihah, Hasan Nudin NurRajput, Vishnu D.Fauconnier, Marie-LaureRaju, RiteshFavas, PauloRakosy‑Tican, ElenaFawley, Karen P.Raliya, RameshFecka, IzabelaRallo, PilarFedorowicz, Joanna DagmaraRamachandran, Sowmya RamachandranFeduraev, PavelRamamoorthy, RengasamyFehér, PálmaRaman, RenukaFekete, AgnesRamegowda, VenkategowdaFeledyn-Szewczyk, BeataRamírez Gil, Joaquín GuillermoFélix, CarinaRamirez, Maria LauraFeller, CarmenRamírez-Bahena, Martha HelenaFeng, HonglinRamírez-Parra, ElenaFentaye, Kassa SemagnRamona Moise, AdelaFerentinos, KonstantinosRamos Cabrer, AnaFereres, AlbertoRamos, JavierFeresin, GabrielaRamos, Patricia A. B.Fernandes, PatríciaRamos, PawełFernandes, PauloRamos, SebastianFernandes-Silva, Anabela A.Ranalli, GiancarloFernandez, EduardoRanieri, PietroFernández, ManuelRapacz, MarcinFernandez-Escobar, RicardoRâpeanu, GabrielaFernández-González, MariaRaposo, MauroFernández-Lorenzo, Juan LuisRasiukevičiūtė, NeringaFernandez-Ondoño, EmiliaRaskina, OlgaFernández-Zamudio, RocíoRasmussen, Hanne N.Fernando, Ilekuttige Priyan ShanuraRašovský, MarekFerrante, AntonioRaspor, MartinFerrante, ClaudioRatet, PascalFerrarezi, RhuanitoRather, IrfanFerreira Dos Santos, Patricia C.Ratiu, AndreeaFerreira, MarceloRato, Ana ElisaFerrer Ayala, María ÁngelesRattenholl, AnkeFerrigo, DavideRaudonė, LinaFerrini, FrancescoRavichandran, MirunaliniFerroni, LorenzoRavin, Nikolai V.Feulner, MartinRawat, SwatiFialová, SilviaRay, SwayamjitFichman, YosefRaynaud, CécileFidan, HafizeRaynor, EdwardField, RichardRayon, CatherineFierascu, IrinaRažná, KatarínaFierascu, Radu ClaudiuRazumova, Olga V.Fikere, MulusewRazzaque, SamsadFíla, JanRebekić, AndrijanaFilek, MariaRebollo-Hernanz, MiguelFilip, MiutaReboredo, HenriqueFilippone, EdgardoRébufa, CatherineFiliz, ErtugrulReda, MałgorzataFilyushin, Mikhail A.Reding, MichaelFin, AndreaRegazzoni, Luca GiovanniFinch, Jonathan T. D.Regni, LucaFinetti-Sialer, Mariella MatildeRego, CeciliaFinkina, EkaterinaReguera, MaríaFinzi, AlbertoRehman, AbdulFiołka, Marta J.Rehman, Attiq UrFiore, Maria CarolaRehrig, ErinFischer, DylanReid, Michael S.Fischer, LukášReig, GemmaFitches, ElaineReigosa, Roger J.Flamini, GuidoReim, StefanieFleituch, TadeuszReis, CarlosFlessner, MichaelReis, PedroFleszar, Mariusz G.Rékási, MárkFletcher, KyleRemizowa, Margarita V.Flieger, JolantaRenčo, MarekFlinn, BarryRenna, LucianaFlorencio, Francisco J.Renner, Matt A. M.Fodor, FerencRenzo, VannaFodor, JozsefResende, Renata SousaFoereid, BenteRestivo, Francesco M.Fogacci, FedericaRestuccia, AlessiaFogarasi, MelindaRetzer, KatarzynaFontaine, FlorenceReveglia, PierluigiFontana, GianfrancoRevilla, PedroFontana, SimonaRewers, MonikaFontes, NatachaRey, LuisFormisano, CarmenReyes López, Joaquin L.Formosa-Dague, CécileReyes, FernandoForni, CinziaRhazi, LarbiForni-Martins, Eliana ReginaRial, CarlosForster, PaulRibeiro, AntónioForti, LucaRibeiro-Barros, Ana I.Forzato, CristinaRicachenevsky, Felipe KleinFouladkhah, AliyarRicci, AntonellaFracchiolla, MarianoRiccioni, ClaudiaFrachisse, Jean-MarieRichardson, Annette C.Francesco, BraccoRichtera, LukášFrancik, RenataRiddick, Eric WellingtonFrancikowski, JacekRiepl, HerbertFrancini, AlessandraRighini, HillaryFranco, JoseRigillo, GiovannaFranco, LourdesRigó, GáborFranić, MarioRihan, HailFrański, RafałRikkerink, ErikFrąszczak, BarbaraRios-Velasco, ClaudioFridman, EyalRisdian, ChandraFriesen, NikolaiRiva, AntonellaFriganović, EmilijaRivera-Vega, LorenFrigau, LucaRiyazuddin, RiyazuddinFriml, JiriRobakowski, PiotrFrolov, AndrejRoberto, Sergio RuffoFrugis, GiovannaRobertson, Alastair W.Fu, QinRobin, ArifFu, XiuminRobustelli Della Cuna, Francesco SaverioFuchs, MarcRoca Fernández, Ana IsabelFujino, TakeshiRocchetti, GabrieleFujita, MasayukiRoccuzzo, GiancarloFujitsuka, NaokiRodamilans, BernardoFujiwara, KazukiRodemann, BerndFukuda, YukiRodiño, A. PaulaFukunaga, KenjiRodrigues, FranciscaFukushima, AtsushiRodrigues, Márcio José De AbreuFurdui, BiancaRodríguez González, ÁlvaroFurini, AntonellaRodríguez Villanueva, JavierFurmańczyk, Ewa M.Rodríguez-Jimenes, GuadalupeFuta, BarbaraRodríguez-Kessler, MargaritaFyhrquist, PiaRodríguez-Lizana, AntonioFyllas, NikolaosRodríguez-López, Carlos E.Gábor, IllésRodriguez-Rajo, Francisco JavierGabriel, Jose LuisRodriguez-Rastrero, ManuelGabriel, RosalinaRodríguez-Rojo, Maria PilarGabriele, MorenaRodríguez-Rosales, M. PilarGabryś, BeataRodríguez-Seijo, AndrésGabur, IulianRodríguez-Solana, RaquelGadhave, KiranRodríguez-Yoldi, María JesúsGaffney, Michael T.Roe, Judith L.Gaglio, MattiasRogers, HilaryGaillochet, ChristopheRohila, Jai S.Gajc-Wolska, JaninaRohn, SaschaGajdoš Kljusurić, JasenkaRojo, JesusGajdošová, AlenaRokaya, MaanGal, Adrian FlorinRolbiecki, RomanGalanidis, AlexandrosRomagosa, IgnacioGalanty, AgnieszkaRoman, KamilGăldean, NicolaeRomano, ElioGalic, AnteRomanov, Georgy A.Galić, NivesRomanowska, ElżbietaGalili, ShmuelRomanyuk, NataliyaGallacher, DavidRomeiras, Maria ManuelGallaher, Sean DavidRomero, IreneGallart, MartaRondanini, Deborah P.Gallo, Francesca RomanaRonga, DomenicoGallo, MonicaRoni, Md Zohurul KadirGalván, AuroraRonsisvalle, SimoneGalvánek, DobromilRonzan, MarilenaGalvez-Valdivieso, GregorioRopelewska, EwaGalvosas, PetrikRosa, AntonellaGamarra, RobertoRosa, RobertGambino, GiorgioRosa-Téllez, SaraGamboa-deBuen, AliciaRoselló, SalvadorGámez-Meza, NohemíRosen, BarryGanatsas, PetrosRoslon-Szerynska, EdytaGancarz, MarekRosnoblet, ClaireGanguly, AnindyaRoß, ChristinaGao, GaryRossello, Josep A.Gao, JunfengRossi, GabriellaGao, MinRotenberg, DorithGao, RuiminRoubelakis-Angelakis, Kalliopi A.Gao, ShiqiangRoussis, IoannisGárate Ormaechea, AgustínRoussos, Peter A.Garcia, AlbaRoux, NicolasGarcía-Gómez, Beatriz EsterRovere, Federica DellaGarcia-Jares, CarmenRowe, NickGarcía-Mauriño, SofíaRoy, SuchismitaGarcía-Murillo, PabloRoy, Swapan KumarGarcía-Otín, Ángel LuisRozalski, MarcinGarcía-Sánchez, SusanaRožanc, JanGarcia-Souto, DanielRu, SushanGarcía-Tomillo, AitorRubino, Federico MariaGardikis, KonstantinosRubinowska, KatarzynaGardiman, MassimoRubio, LourdesGardiner, JasonRubio-Cabetas, M. J.Garganese, FrancescaRubio-Casal, Alfredo EmilioGargano, DomenicoRucińska-Sobkowiak, RenataGarhwal, Abhimanyu SinghRudell, David R.Garmash, Elena V.Rudnóy, SzabolcsGarofulić, IvonaRudrabhatla, SairamGarrido, AdriánRuelland, EricGarrido, CarlosRuge-Wehling, BrigitteGarzoli, StefaniaRugienius, RytisGaši, FuadRui, YukuiGaskin, JohnRuíz, LeonorGasparatos, DionisiosRuiz-Albert, JavierGa̧stoł, MaciejRuiz-Garcia, LeticiaGasulla Vidal, FranciscoRuíz-May, ElielGates, ColinRumbos, Christos I.Gatesy, John E.Rumyantseva, NatalyaGaudio, LucianoRungis, DainisGavarane, Inese GavaraneRunion, George BrettGavazzi, GiuseppeRupa, Esrat JahanGaweł-Bęben, KatarzynaRusinowski, SzymonGawroński, JacekRusso, AlessandraGazza, LauraRusso, DanielaGdula-Argasińska, JoannaRustia, Dan Jeric ArcegaGe, XiaoweiRustioni, LauraGebauer, GerhardRusu, DanielaGecheva, GanaRusu, TeodorGeddes-McAlister, JenniferRutkowska, BeataGediga, KrzysztofRutkowska, JaroslawaGelmini, FabrizioRužičić, StankoGeng, QifangRyant, PavelGenovese, GiuseppaRybczyńska-Tkaczyk, KamilaGentile, AlessandraRybka, KrystynaGentile, CarlaRychel, SandraGeoffriau, EmanuelRyś, MagdalenaGeorgia, NtatsiRyšlavá, HelenaGeorgieva, MargaritaRyss, AlexanderGeorgiou, Christos D.Ryu, JaihyunkGerbeau-Pissot, PatriciaRzemieniec, JoannaGerdol, RenatoSabatier, SylvieGere, AttilaSabatino, LeoGerm, MatejaSabbadini, SilviaGermano, Maria PaolaSabella, ErikaGesell, AndreasSabovljevic, MarkoGevrenova, RenetaSache, IvanGhanotakis, Demetrios DemetriosSaddala, Madhu SudhanaGhiasi, Seyed MojtabaSadeghifar, HasanGhigliotti, LauraSadgrove, NicholasGhilardi Lago, João HenriqueSadhukhan, AyanGhimire, BalkrishnaSaftić Martinović, LaraGhimire, Bimal KumarSager, ManfredGhiselli, LisettaSagor, G. H. M.Ghodake, GajananSah, Saroj KumarGhosal, SambuddhaSaha, BedabrataGhose, KaushikSaha, SubhasishGhosh, SoumitaSaha, Sushanta KumarGiacomo, MeleSahoo, NirakarGiamperi, LauraSahuquillo, Elvira BalbuenaGianinetti, AlbertoSaifutdinov, Bulat R.Giannakou, IoannisSaiz-Fernández, IñigoGiannuzzi, LedaSajeva, MaurizioGibczyńska, MarzenaSajnaga, EwaGieczewska, KatarzynaSakai, AtsushiGiertych, MarianSakamoto, AyakoGietler, MartaSakamoto, MasahiroGiffard, BriceSakamoto, MasaruGifford, Miriam L.Sakamoto, TakuyaGilca, MarilenaSala, FlorinGiles, GregorySalamon, IvanGil-Pelegrín, EustaquioŠalamún, PeterGins, MuratSalanki, KatalinGiordano, MariaSalánki, KatalinGiorni, PaolaSalata, AndrzejGiouri, KaterinaSalazar, Juan A.Giovanneti, ManuelaSalazar, Juan RodrigoGiovino, AntonioSalbitani, GiovannaGismondi, AngeloSalbitano, FabioGiuffrè, Angelo MariaSaleh-E-In, Md. MoshfekusGiuggioli, Nicole RobertaSales, EsterGkizi, DanaiSalimonti, AmeliaGladan, Živana NinčevićSalmeri, Cristina MariaGladish, Daniel K.Salopek-Sondi, BrankaGladkikh, IrinaSalud, SerranoGładyszewska, BożenaSalvi, PrafullGlasa, MiroslavSamac, DeborahGlazińska, PaulinaSamakovli, DespinaGligor, FeliciaSamalova, MarketaGnanasekaran, PrabuŠamec, DunjaGniazdowska-Piekarska, AgnieszkaSamigullin, Tahir H.Gobin, IvanaSamira, RozalynneGoddard, Mary-LorèneSampaio, Bruno LeiteGodena, SaraSampaio, Geni RodriguesGodlewska, PatrycjaSamuoliene, GiedreGoggin, Danica E.Sánchez Ballesta, María TeresaGogo, Elisha OtienoSánchez Del Pino, Manuel M.Gogolev, YuriSánchez, Diego HernánGoia, Irina GabrielaSánchez-Rodríguez, Antonio RafaelGolan, KatarzynaSancho Dos Santos, Carla SofiaGolczyk, HieronimSandalio, Luisa MaríaGoldsmith, MosheSangiovanni, EnricoGolia, EvangeliaSannomiya, MiriamGolinelli, Marie-PierreSano, SatoshiGolob, AleksandraSantamaria, ÓscarGolokhvast, KirillSantangelo, AnnalisaGolonka, IwonaSantilli, ElenaGolsteyn, Roy M.Santos López, Jorge ArturoGolubkina, NadezhdaSantos, Carla S.Gombojav, AriunboldSantos, MilaGomes, Carlos MartinsSaracchi, MarcoGomes, FernandaSaratale, GaneshGomes, FilomenaSarigu, MarcoGomes, SóniaSarkar, DibyenduGomez De Barreda Ferraz, DiegoSasal, YamilaGomez Garay, AranchaSaska, PavelGómez López, PedroSatake, MasayukiGómez, Christian R.Satish, LakkakulaGómez, Rodrigo L.Sato, HikaruGómez-Barrero, SusanaSatoh, ShigeruGómez-Martín, CristinaSatyal, PrabodhGómez-Mena, ConcepciónSava, Camelia SandGomez-Merino, FernandoSavchenko, Tatyana V.Gomez-Silva, BenitoSavoi, StefaniaGomis-Cebolla, JoaquinSavoie, Jean-MichelGonçalves, Ana C.Savvidou, MariaGonçalves, PedroSawicka, BarbaraGonçalves-de-Albuquerque, Cassiano FelippeSawicki, JakubGonda, SándorSawicki, RafalGong, HaijunScaccini, DavideGontier, EricScala, EmanueleGonzález Andújar, José LuisScalschi, LoredanaGonzalez Torralva, FidelScarafoni, AlessioGonzález, AlbertoScarano, AureliaGonzález, BernardoScarpa, Grazia MariaGonzález, José A.Scepanovic, MajaGonzalez, JuanSchaller, JörgGonzález, LuísScharff, LarsGonzalez-Benito, Maria ElenaSchenk, JohnGonzález-Cortazar, ManasésScherm, HaraldGonzález-Delgado, Jose A.Scherrer, DanielGonzález-De-Peredo, Ana V.Schiavon, MichelaGonzález-Fontes, AgustínSchicchi, RosarioGonzález-Mas, M. CarmenSchiermeyer, AndreasGonzález-Minero, Francisco JoséSchinella, Guillermo R.Gonzali, SilviaSchinnerl, JohannGoodrich, Gregory B.Schlossberg, Maxim J.Gora-Sochacka, AnnaSchmidt, AnjaGori, AntonellaSchmidt, DominikGorpenchenko, Tatiana Y.Schmidt, MathiasGorshkov, VladimirSchmitt-Keichinger, CorinneGorshkova, Tatyana A.Schmull, MichaelaGórska, AgataSchoberer, JenniferGorska, Ewa BeataSchreel, JeroenGórska, Ewa BeataSchreiber, Karl J.Gospodarek, JaninaSchrick, KatrinGostin, Irina NetaSchröder, Fritz-GeraldGotor, Alicia AyerdiSchubert, AndreaGötz, Klaus-PeterSchubert, IngoGouguet, PaulSchuster, MichaelGoulao, LuisSchymura, StefanGouveia, Alexandra M.Sciubba, FabioGoyali, Juran C.Scortichini, GiampieroGozdowski, DariuszScrano, LauraGrabarczyk, MałgorzataSearcy, ChristopherGrabau, Larry J.Sebastiana, MónicaGracia-Romero, AdrianSeger, AndreasGraciet, EmmanuelleSeghal, AkankshaGrammatikopoulos, GiorgosSehgal, DeepmalaGranica, SebastianSeitz, BjoernGrassi, FabrizioSekiewicz, KatarzynaGray, JohnSekine, MasamiGrdiša, MartinaSeleiman, Mahmoud F.Greco, CarloSelvakesavan, Rajendran KamalabaiGreco, NicolaSelvaraj, ArokiyarajGreen, David R.Semeniuc, Cristina AnamariaGreer, DennisSempruch, CezaryGreisen, Per JrSennikov, AlexanderGresta, FrancescoSenze, MagdalenaGrewe, FelixSeo, DaisukeGrieco, FrancescoSeo, ShigemiGriesser, MichaelaSeppelt, RodGriffith, M. PatrickSera, BozenaGriga, MiroslavŞeremet, Oana CristinaGrigore, Marius-NicusorSergiev, IskrenGrillet, LouisSergio, LucreziaGrillo, RenatoSerio, FrancescoGrishko, VictoriaSerra, PedroGrivalský, TomášSerra, StefanoGródek-Szostak, ZofiaSerralheiro, Maria LuísaGromova, MarinaSerrano, RitaGrones, PeterSestras, Adriana F.Grossi, DanieleSestras, RaduGrossman, JakeSetzer, William N.Grossmann, KatjaSevastre, BogdanGrover, SajjanSevcovicova, AndreaGrubešić, Renata JurišićSeverns, Paul MGruden, KristinaSewordor, DavidGrulova, DanielaSgaragli, GiampietroGrynkiewicz, GrzegorzSgarbi, ElisabettaGrzebelus, EwaSgherri, CristinaGrzegorczyk-Karolak, IzabelaShaaf, SalarGrzybowski, MirosławShafiei, RezaGu, LipingShafiq, SarfrazGu, LiuqiShah Jahan, MohammadGuarino, FrancescoShahid, SaimaGuarino, SalvatoreShahinnia, FahimehGuerineau, FrançoisShaik, Haq AbdulGuerre, PhilippeShaik, RaziaGuerrina, MariaShaltout, Kamal HussienGuevara Garcia, Angel ArturoShao, ShengxiGuevara, RamónSharma, AjayGugliuzza, GiovanniSharma, AnamikaGuiamet, Juan-JoséSharma, AnketGuignard, GaëtanSharma, GyanGuihur, AnthonySharma, KavitaGuillén-Bejarano, RafaelSharma, LavGujas, BojanSharma, NiharikaGullì, MariolinaSharma, PritiGunaje, JayaramaSharpe, ShaunGundel, PedroShchennikova, AnnaGunjača, JerkoShcherban, Andrey B.Guo, JingshuShelake, Rahul MahadevGuo, MingquanShelp, Barry J.Guo, QiShen, JianqiangGupta, ParulShen, Szu-ChuanGupta, RaviSheng, RuilongGupta, Sanjay K.Sheppard, PaulGururani, Mayank AnandSherstneva, OksanaGurusamy, RamanShevock, JamesGurusinghe, SaliyaShibaeva, Tatjana G.Gustafson, Danny J.Shilev, StefanGutaker, RafalShimakawa, GingaGutekunst, KirstinShimelis, HusseinGutiérrez Mañero, Francisco JavierShimomura, HirofumiGutiérrez, SalvadorShin, DongjinGutiérrez-Gamboa, GastónShinano, TakuroGutiérrez-Gutiérrez, CarlosShinomura, TomokoGutiérrez-Méndez, NéstorShinozaki, JunichiGutiérrez-Miceli, FedericoShishova, Maria F.Guzman, CarlosShneyer, VictoriaGuzmán, MiguelShokat, SajidGuzzetti, LorenzoShtein, IlanaHa, Bo-KeunShukla, MukundHabili, NuredinShukla, Pushp SheelHaelewaters, DannyShumilina, Daria V.Hafez, Elsayed E.Shuyskaya, ElenaHage-Ahmed, KarinSiadjeu, ChristianHagemann, MartinSiatkowski, IdziHaigh, AnthonyŠic Žlabur, JanaHájková, LenkaSicard, PierreHakeem, Khalid RehmanSiddiqui, Manzer H.Hála, MichalSieczko, LeszekHaliniarz, MałgorzataSielemann, KatharinaHalitschke, RaykoSiemianowski, OskarHallmann, EwelinaSieprawska, ApoloniaHamberg, LeenaSierka, EdytaHambuckers, AlainSierra, ArturoHamerlynck, ErikSigdel, Shalik RamHamułka, JadwigaSilas Fernandes, EtoHan, Yeon SooSilber, AvnerHanaka, AgnieszkaŠiler, BranislavHanba, Yuko T.Šilha, DavidHančević, KatarinaSilva, Amelia MHancock, Charles NathanSilva, Anderson TadeuHancock, JohnSilva, IvanovitchHancu, GabrielSilva, Maria HelenaHanganu, DanielaSilva, SóniaHano, ChristopheSilva-Filho, Marcio C.Hanslin, Hans MartinSilvestri, CristianHanus-Fajerska, Ewa JoannaSima, Nicusor-FlaviusHardion, LaurentSima, Rodica MariaHarhar, HichamSimard, Marie-JoséeHark, Amy T.Simirgiotis, MarioHarkess, RichardSimlat, MagdalenaHarms, Nathan E.Simm, StefanHarrington, Kerry C.Simões, FernandaHarris, DavidSimon, AnneHarris, LarrySimon, Maria RosaHartman, MariuszSimone, NicolaHartman, SjonSinapidou, EvangeliaHartmann, AntonSingh Tuli, HardeepHartung, JensSingh, Bal RamHarvengt, LucSingh, KashmirHarvey, PatriciaSingh, Ritesh KumarHasanuzzaman, MirzaSingh, Rupesh KumarHase, YoshihiroSingh, SurjitHasegawa, MorifumiSinjushin, Andrey A.Hasenstein, KarlSipos, LászlóHashem, AbulSirko, AgnieszkaHashimoto, KenjiSiudem, PawełHasiów-Jaroszewska, BeataSivanesan, IyyakkannuHasnagas, Nikolaos D.Sivarajan, Sajeevan RadhaHassan, ErrolSjöstedt, CarinHassan, Sherif T. S.Skała, EwaHassani, Mohamed-AmineSkejić, SandaHastwell, AprilSkoczylas, ŁukaszHatteland, Bjørn ArildSkorupa, MonikaHatzilazarou, StefanosSkouras, Panagiotis J.Hausladen, HansSkrypnik, LiubovHavaux, MichaelSkrzypek, EdytaHawes, CathySkutnik, EwaHawkesford, Malcolm J.Skuza, LidiaHawrylak-Nowak, BarbaraŚlesak, HalinaHe, BaoyeŚlipiko, MonikaHe, GuohaoŚliwińska-Wilczewska, SylwiaHe, JieŚliwka, MałgorzataHe, XinqiangSlováková, ĽudmilaHealey, AdamSmaranda, CameliaHearn, DavidSmedbol, ÉliseHeckmann, StefanSmeriglio, AntonellaHefferon, KathleenŚmigielski, Krzysztof B.Hegazy, Mohamed-Elamir F.Smilkov, KatarinaHegedűsová, AlžbetaSmith, Duncan D.Heinrichs, SteffiSmith, Kevin T.Hejna, MonikaSmoleń, SylwesterHelton, Rebekah R.Smolinska, BeataHemkemeyer, MichaelSmutná, MarieHemp, Andreas J.Smykal, PetrHendricks, JosephSnopek, LukasHeneberg, PetrSoares, CristianoHenke, RobertoSobiech, ŁukaszHenrik Nilsson, RolfSofo, AdrianoHenry, AjunaSofy, Ahmed R.Hepworth, Shelley R.Sogorb Sánchez, Miguel ÁngelHerde, MarcoSoica, CodrutaHernández, FranciscaSokólski, MateuszHerrbach, EtienneSokovic, MarinaHerrera, HectorSolanki, ShyamHerrera, MartaSolano, FranciscoHerrero, BaudilioSolcan, CarmenHesami, MohsenSolhaug, Knut AsbjørnHess, MichaelSolomon, Juan K. Q.Hessing-Lewis, Margot L.Solovyev, AndreyHewedy, OmarSoltabayeva, AigerimHewson, JenniferSolti, ÁdámHidalgo, OrianeSoltys, KatarinaHilario, ElenaSomerville, Gayle J.Hilbeck, AngelikaSomeya, NobutakaHill, SandraSommaggio, DanieleHilliou, FrederiqueSon, Chang-HwanHimanen, KatriSon, Ki-HoHimuro, ChihiroSonah, HumiraHinojosa, LeonardoSong Bi, ChenHinz, HarietSong, Jun-HoHirata, ToshiyukiSong, Kwan-JeongHiroko, HayamaSong, QinHiroyuki, KobayashiSongstad, D. D.Hlihor, Raluca MariaSonnino, AndreaHnatuszko-Konka, KatarzynaSopeña-Torres, SaraHo, Shin-LonSorgonà, AgostinoHoang, NamSoria, Ana CristinaHoang, Phuong T. N.Sosa-Hernández, MoisésHocart, CharlesSosnowska, KatarzynaHochwagen, AndreasSoteriou, GeorgeHodel, Richard G. J.Sottile, FrancescoHodge, SimonSõukand, RenataHodges, MichaelSoundararajan, PrabhakaranHoffmann, Lucia VieiraSousa, Maria C.Höglind, MatsSouto, Consuelo PaolaHolb, ImreSouto, DanielHolík, LadislavSouza, Gustavo MaiaHolland, Marjorie M.Sowa, IreneuszHolme, IngerSpanò, CarmelinaHolonec, LiviuSpasov, Alexander A.Holt, Scott M.Sperdouli, IlektraHolub, PetrSpicer, Michelle EliseHolubec, VojtechSpirchez, CosminHonermeier, BerndSpochacz, MartaHong, Chi-EunSpornberger, AndreasHooykaas, PaulSprio, Andrea ElioHorel, ÁgotaŠpundová, MartinaHori, KiyosumiSrivastava, Dhruv AdityaHorn, PatrickSroka-Bartnicka, AnnaHorstman, AnnekeStachiv, IvoHorta, CarmoStadlbauer, PetrHortigón-Vinagre, MaríaStahl, EliaHorvat, DanielaStahl, Judith M.Horvat, Katarina PolajnarStahl, Randal S.Horvath, DavidŠtajner, NatašaHossain, ShahdatStalenga, JarosławHosseini, Seyed AbdollahStancheva, IraHouliston, GaryStanek-Tarkowska, JadwigaHowell, PhilStaniak, MariolaHrabal, RichardStanica, FlorinHradil, PavelStankiewicz-Kosyl, MartaHrckova, GabrielaStanković, JelenaHristov, BiserŠtarha, PavelHrivnák, RichardStarowicz, MałgorzataHronková, MarieStasiak-Różańska, LidiaHsu, Fu-ChiunStaszak, Aleksandra MariaHsu, Ssu-WeiStaszek, PawelHsu, Yu-ChiaStavolone, LiviaHu, ChungchiŞtefan, Daniela SiminaHu, HaifeiŠtefániková, JanaHu, HaixiaoStefanou, StefanosHu, JieSteger, GerhardHu, KainingSteinbrecher, RicardaHu, QiangStephenson, ChristianHu, QingStępień, ŁukaszHu, TaoStępiński, DariuszHu, YanSteuer, ChristianHua, KhuongStevenson, DennisHuang, Guan-JhongStobiecki, MaciejHuang, JiStodart, BenHuang, JianliangStojałowski, StefanHuang, JianzhiStojković, DejanHuang, MengyuanŠtolfa, IvnaHuang, Nen-FuStoyneva-Gärtner, MayaHuang, Pei-ChengStpiczyńska, MałgorzataHuang, ShouguangStreit, KethrinHuché-Thélier, LydieStroe, Silviu-GabrielHuchzermeyer, BernhardStrojny-Cieslak, BarbaraHuff, David R.Struck, ChristineHughes, GabrielStrumia, SandroHuh, JungmooStrzałek, MałgorzataHulin, MichelleStrzemski, MaciejHumbeck, KlausStudnicki, MarcinHunter, CharlesSu, HuananHusgafvel, RoopeSu, JianbinHussain, HidayatSubbotin, Sergei A.Hussain, Muhammad IftikharSubramani Paranthaman, BalasubramaniHussain, SaddamSubramanian, SowmyalakshmiHwang, Yoon-JungSudheeran, Pradeep KumarI. Kalinin, VladimirSugano, Shigeo S.Iacopetta, DomenicoSugawara, AkihiroIametti, StefaniaSugier, PiotrIbrahim, IskanderSuh, Hyung JooIchitani, KatsuyukiSuharoschi, RamonaIelciu, IrinaȘuică-Bunghez, RalucaIevinsh, GedertsSujka, MonikaIglesias, Carlos A.Sujkowska-Rybkowska, MarzenaIgliński, BartłomiejSukhorukov, Alexander P.Ignatov, AlexanderSukhova, EkaterinaIgnatov, M. S.Sukumaran, SunithaIkkonen, Elena N.Sukunimetön, PriyankaIlia, GheorgheSulborska, AnetaIllés, ÁrpádSułkowska, MałgorzataImamura, SousukeSumalan, Radu LiviuImran, Qari MuhammadŠumberová, KateřinaIndreica, AdrianSumitomo, KatsuhikoInnangi, MicheleSumorok, BeataInostroza-Blancheteau, ClaudioSun, HongyongInouye, David W.Sundh, IngvarIoannis, GiannakouSung, JwakyungIoos, RenaudSungthong, RungrochIoras, FlorinSunseri, FrancescoIorizzi, MariaSun-woo, ChungIovene, MarinaŠupljika, FilipIovieno, PaoloSuprun, Andrey R.Ipsilandis, ConstantinosSur, IoanaIrfan, MohammadSurmacz, LilianaIrving, LouisSurówka, EwaIsayenkov, StanislavSushkova, SvetlanaIseki, MineoSusila, HendryIshikawa, ShokoŠuštar-Vozlič, JelkaIslam, Md. RafiqulSut, StefaniaIslam, Md TariqulSutherland, Hamish S.Islam, Md TohidulSuzuki, KatsuhikoIslam, MonirulSuzuki, RyuichiroIsola, DanielaŠvedienė, JurgitaItabashi, EtsukoSvitková, IvanaIto, ShinsakuŠvubová, RenátaIturriaga, GabrielSwiecilo, AgataIvanov, RumenSwiezewska, EwaIvanova, LarissaSykłowska-Baranek, KatarzynaIvanović, MilenaSykora, DavidIvanovska, NinaSymanowicz, BarbaraIwai, AtsushiSynowiec, AgnieszkaIwashina, TsukasaSytar, OksanaIzquierdo, JordiSytykiewicz, HubertIzydorczyk, GrzegorzSzabo, KatalinJabran, KhawarSzala, LaurencjaJacobo-Velazquez, DanielSzczepanek, MałgorzataJacygrad, EwelinaSzczepaniak, JanJaganjac, MoranaSzentgyörgyi, HajnalkaJagosz, BarbaraSzewczyk, AgnieszkaJahandideh, ForoughSzewczyk, KatarzynaJahnke, Gizella GyorffyneSzilasné Jósvai, Júlia KatalinJain, Shri MohanSzopinski, MichałJaiswal, Amit K.Szostek, MałgorzataJakab, GergelySztanke, MałgorzataJakaria, Md.Szulc, PiotrJakovljevic, MartinaSzumny, AntoniJakse, JernejSzymanowska-Pułka, JoannaJakubczyk, AnnaSzymańska, GrażynaJakubska-Busse, AnnaSzymańska, MagdalenaJames, ArnoneSzymański, MarcinJamroz, ElżbietaSzymura, MagdalenaJan, Fuh-JyhTabacchioni, SilviaJan, HaberleTacconelli, StefaniaJana, LibantováTacconi, GianniJancikova, SimonaTaddei, FedericaJanczukowicz, WojciechTaglienti, AnnaJanda, TiborTahir, AmmarJang, Tae-SooTahir, JibranJanicka-Russak, MałgorzataTahjib-Ul-Arif, MdJaniszewska-Turak, EmiliaTakagi, DaisukeJanja, KristlTakahashi, MisaJankowska, EwelinaTakaichi, ShinichiJanni, MichelaTambone, FulviaJansen, StevenTamburino, RacheleJanušauskaitė, DaivaTamez-Guerra, PatriciaJanuskaitiene, IrenaTan, Ek HanJaradat, NidalTanase, CorneliuJaramillo-Morales, Osmar AntonioTančinová, DanaJarecki, WacławTanemossu Fobofou, Serge AlainJarolmasjed, SanazTang, BuyunJaromír, LukavskýTang, QingyunJarošová, JanaTapia, Alejandro A.Jarret, RobertTarakanov, IvanJarzebski, MaciejTarkowska-Kukuryk, MonikaJasińska, AgnieszkaTARRAGO, LionelJastrzębska, MagdalenaTasic, LjubicaJasutiene, InaTasinov, OskanJaumot, JoaquimTatarinov, FedorJaworska, HannaTavares Carvalho, José CarlosJazvinšćak Jembrek, MajaTavares, Maurício Temotheo TemotheoJedmowski, ChristophTayade, RupeshJedrejek, DariuszTchórzewska, DorotaJȩdrszczyk, ElżbietaTedeschi, PaolaJelaska, SvenTedesco, IdoloJenkins, ColinTegli, StefaniaJensen, KaiTeige, MarkusJeong, Dong-HoonTeixeira, NatérciaJeong, Rae-DongTellgren-Roth, ChristianJeranyama, PeterTello Moro, JavierJerković, IgorTello, JavierJeszka-Skowron, MagdalenaTéoulé, EvelyneJiang, GuoxiangTermolino, PasqualeJiang, JunyeTeruko, KonishiJiang, Shu-YeTesta, AntoninoJibran, RubinaThalmann, MatthiasJiménez Gómez, Pedro A.Thanos, CostasJiménez-Araujo, AnaThekkiniath, JoseJiménez-Ruiz, JesúsThilmony, RogerJin Cheol, KimThiruvengadam, MuthuJindo, KeijiThomas, JohnJiri, VrbaThomas, RichardJo, Ick-HyunThomas, StefanJoachimiak, AndrzejThor, KathrinJócsák, IldikóThorlby, Glenn J.Johannes, WöstemeyerThorpe, Michael RobertJohn Jervis, PeterThussagunpanit, JutipornJohnson, DonnTibpromma, SaowaluckJones, Meriel G.Tikhonova, IrinaJordheim, MonicaTirado-Corbalá, RebeccaJorrin-Novo, Jesus V.Tirillini, BrunoJoshi, Pushpa RajTit, Delia MirelaJoulain, DanielTiwari, ArjunJuárez-Maldonado, AntonioTiwari, SudeepJubeau, SébastienTjamos, SotirisJucan, DenisaTkachenko, KirillJudzentiene, AstaTkaczuk, CezaryJullian, NathalieTlelo-Cuautle, EstebanJunaid, MuhammadTo, Kin-YingJuncan, Anca MariaTodaka, DaisukeJúnior, Manoel Teixeira SouzaTodd, JamesJunior, Mario LiraToderi, MarcoJurado, Macarena M.Toderich, KristinaJura-Morawiec, JoannaTodorova, DessislavaJurić, SlavenTokarz, BarbaraJurinjak Tusek, AnaTomaszewski, DominikJurkonienė, SigitaTomaz, IvanaJuśkiewicz, JerzyTombesi, SergioKabir, Mohammad FaujulTomiczak, KarolinaKačániová, MiroslavaTomohito, SanoKacjan Maršič, NinaTopal, Ali OsmanKafarski, PawelTopalovic, OliveraKafkaletou, MinaToporowska, MagdalenaKagan, IsabelleTorta, LivioKageyama, HakutoTóth, BrigittaKajiya-Kanegae, HiromiToth, PeterKakabouki, IoannaTouraine, BrunoKalaji, Hazem MohamedTown, JenniferKalbarczyk, RobertToyota, KoikiKalemba, Ewa M.Tramontini, SaraKalendar, RuslanTran, Phu-TriKalisz, StanisławTraoré, Amidou S.Kalle, RaivoTrdan, StanislavKalló, GergőTreder, KrzysztofKaló, PéterTremblay, Émilie D.Kaltsa, OlgaTremblay, RaymondKamal, Khaled Y.Tres, Marcus ViniciusKamiloglu, SenemTrifan, AdrianaKamiya, KoichiTrifilò, PatriziaKamle, MadhuTrigiano, RobertKamzolova, SvetlanaTripathi, Jaindra NathKanagendran, ArooranTroccoli, AlbertoKanavillil, NandakumarTrognitz, FriederikeKancharla, PapireddyTroitsky, AlexKandagalla, ShivanandaTroncoso-Ponce, AdrianKandel, Prem PrasadTrovato, MaurizioKanekatsu, MotokiTrucillo, PaoloKanellos, PanagiotisTruong, Hai AnKanetis, LoukasTruzzi, FrancescaKang, Kwon KyooTsafack, NoellineKang, Li-WeiTsai, Chen-ChiKang, Woo HyunTsai, Jen-ChiehKang, Yang JaeTsai, Mei-LinKano, NaokiTsai, Po-JungKao, Chung-FengTsakanikas, PanagiotisKao, Shao-HsuanTsaniklidis, GeorgiosKapazoglou, AlikiTseng, Chih-HuaKapczyńska, AnnaTsianou, Mariana A.Kappachery, SajeeshTsikou, DanielaKappel, NoemiTsim, KarlKapusta, MalgorzataTsouknidas, AlexanderKaragiannis, EvangelosTsouvaltzis, PavlosKaramaouna, FilitsaTsubota, HiromiKaranastasi, EiriniTugizimana, FideleKarcz, DariuszTuleja, MonikaKardos, JuliannaTullius Scotti, MarcusKärenlampi, Petri P.Tumialis, DorotaKarkanis, AnestisTünde, JurcaKarlíčková, JanaTundis, RosaKarpavičienė, BirutėTundo, SilvioKarpova, Olga V.Tung, Chih WeiKarsai, IldikoTurab Raza, SyedKartashov, AlexanderTurkiewicz, Igor PiotrKarunarathna, SamanthaTvrdá, EvaKasahara, Ryushiro D.Twardowska, IrenaKashman, YoelTychkov, Ivan I.Kasianov, Artem S.Tyczewska, AgataKasiotis, KonstantinosTylova, EditaKatam, RameshTylová, EditaKateris, DimitriosTymoszuk, AlicjaKato, Massuo JorgeTyteca, DanielKatsenios, NikolaosTzanova, MilenaKatz, OfirTzortzakakis, EmmanuelKaur, RimaljeetTzortzakis, NikosKaushik, PrashantUberti, FrancescaKaveh, HamedUhde-Stone, ClaudiaKawakami, SusumuUlloa, HugoKayano, Shin-ichiUmber, MarieKelleher, ColinUndersander, DanKelley, JoshuaUpadhyay, Rakesh K.Kenfack, DavidUpadhyay, SantoshKerchev, PavelUrbaniak, JacekKereša, SnježanaUrlić, BranimirKern, RamonaUrsache, RobertasKeutgen, NorbertVaida, IoanaKhairullin, RamilVainonen, JuliaKhaliluev, Marat R.Vaira, Anna MariaKhallouki, FaridValdés López, OswaldoKhan, HabibValenzuela, HectorKhan, MerajuddinValenzuela, Juan LuisKhan, Nafees A.Valiuskaite, AlmaKhan, Naqib UllahValletta, AlessioKhapugin, Anatoliy A.Valli, Adrian AlejandroKhodaeiaminjan, MortazaValoroso, Maria CarmenKhoo, CheangValverde, ClaudioKhrustaleva, LiudmilaVan Cutsem, PierreKiefer, PatrickVan Deenen, NicoleKiełbowicz-Matuk, AgnieszkaVan Den Broeck, LisaKieliszek, MarekVan Der Linde, KarinaKiełkowska, AgnieszkaVan Der Maesen, Laurentius Josephus GerardusKikuchi, HaruhisaVan Der Nest, Magriet A.Kilanowicz, AnnaVan Der Werff, HenkKilli, DilekVan Dobben, HanKim, Chuk YoungVan Dyke, MichaelKim, DongjinVan Emon, Jeanette M.Kim, Ho BangVan Heusden, Adriaan W.Kim, HoyeunVan Klink, John WilliamKim, Hyoung TaeVan Straalen, Nico M.Kim, Hyun UkVan Tuyl, JaapKim, Hyun-SoonVan Wuytswinkel, OlivierKim, In-JungVandebroek, InaKim, InyongVandenbussche, FilipKim, Jae KwangVannini, AndreaKim, JaeyoonVantarová, KatarínaKim, Jung SunVardanega, RenataKim, Jung SungVarela, Maria CarolinaKim, JunheonVarga, IvanaKim, Kyung-MinVargas-García, María Del CarmenKim, Min-SukVargas-Murga, LilianaKim, SangtaeVaris, SailaKim, Sang-TaeVarnagiryte-Kabasinskiene, IvetaKim, Seung HyunVarvaresou, AthanasiaKim, Seung-HyunVassallo, AntonioKim, Sung KyeomVaverková, Magdalena DariaKim, Sung-RyulVelada, IsabelKim, Tae-HwanVelasco, LeonardoKindlmann, PavelVeldtman, RuanKirály, GergelyVella, Filomena MonicaKirály, LórántVenkatesh, JelliKirby, ChrisVennapusa, AmaranathaKiselev, Konstantin V.Veranic, PeterKiss, RékaVerdeguer, MercedesKiss, TivadarVerdú, JoséKissling, JonathanVeremeichik, G. N.Kissoudis, ChristosVergeldt, FrankKitazawa, HiroakiVerger, StéphaneKizis, DimosthenisVergine, MarziaKjelgren, RogerVerle, JoseKlamkowski, KrzysztofVerma, SubodhKlasa, AndrzejVernet, PhilippeKlavina, DartaVeronesi, FedericoKlavins, LinardsVeronico, PasquaKlensporf-Pawlik, DorotaVershinin, Alexander V.Klepikova, Anna V.Verstraeten, IngeKlikocka, HannaVetere, AmedeoKlíma, MiroslavViana, Inés G.Klimek-Chodacka, MagdalenaViapiana, AgnieszkaKlimek-Kopyra, AgnieszkaVicaș, Laura GrațielaKlimešová, JitkaVicente, Filipa A.Klipcan, LironVicente, OscarKlochkov, SergeyVidican, RoxanaKnupp, GerdVieira, Maria Lucia CarneiroKo, Horng-HueyVieira, PauloKo, KisungVielba, Jesús MaríaKo, Swee-SuakVijverberg, KittyKoba, TakatoVikram, PrashantKobae, YoshihiroVilagrosa, AlbertoKobayashi, KappeiVilanova, EmilioKobayashi, KoichiVílchez, Juan IgnacioKobayashi, Natsuko I.Viljevac Vuletić, MarijaKoce, Jasna DolencVillalobos Escobedo, Jose ManuelKocsy, GáborVillani, MariacristinaKocurek, MaciejVillano, CliziaKodama, HiroakiVillegas, DolorsKodym, AndreaVillena, GrettyKoehler, HartmutVinci, GiulianaKohyama, NorikoVincze, EvaKokoska, LadislavVining, KellyKokotkiewicz, AdamVishnyakova, MargaritaKoksharova, Olga A.Visioli, GiovannaKollárová, KarinViškelis, PranasKollers, SonjaViskup, RichardKollmann, JohannesVita, FedericoKolmos, ElsebethVitti, AntonellaKołodyńska, DorotaViuda-Martos, ManuelKołodziejczyk-Czepas, JoannaVives Cobo, CristinaKołton, AnnaVlachonasios, KonstantinosKomínek, PetrVlainić, JosipaKomis, GeorgeVogelweith, FannyKomorowska, BeataVoigt, DagmarKonarska, AgataVokurka, AlešKończak, MagdalenaVolk, GayleKong, LishengVolkov, Alexander G.Kong, ZhiqiangVollmann, JohannKonieczynski, PawelVončina, DarkoKonishi, MasaakiVoothuluru, PriyaKono, MasaruVoronova, AngelikaKononenko, Neonila V.Voxeur, AlineKonôpka, BohdanVrsic, StankoKonopka, IwonaVu, Danh C.Konrad, HeinoVurukonda, Sai Shiva Krishna PrasadKonrádová, HanaVuts, JozsefKontunen-Soppela, SariWadl, PhillipKönyves, KálmánWagensommer, Robert PhilippKooliyottil, RinuWajs-Bonikowska, AnnaKopeć, PrzemysławWalker, Robert J.Kopjar, MirelaWalling, Jason G.Koppolu, RaviWalter, MonikaKorać, PetraWang, Chao-MinKorakaki, EvangeliaWang, DongHaoKoramutla, Murali KrishnaWang, HuanzhongKormutak, AndrejWang, Hui-minKorobova, Alla V.Wang, LeiKorotkova, NadjaWang, Ya-NanKorpelainen, HelenaWang, ZhuangjiKosakivska, Iryna V.Wangchuk, PhurpaKosakowska, OlgaWannicke, NicolaKosmala, ArkadiuszWarchoł, MarzenaKosmala, MonikaWase, NishikantKosová, KláraWassenegger, MichaelKostecka-Gugala, AnnaWatanabe, AtsushiKostić, Aleksandar Ž.Weeden, Norman FKostyn, KamilWehling, PeterKotenkova, ElenaWehr, BernhardKothari, Shanker LalWei, JunKotnik, PetraWeibull, JensKotsopoulos, ThomasWeiss-Penzias, PeterKottmann, LorenzWen, Chi-KuangKoubouris, GiorgosWen, JiangqiKoudounas, KonstantinosWeryszko-Chmielewska, ElzbietaKouli, KaterinaWestphal, AndreasKováčik, JozefWhent, MonicaKováčik, PeterWidemann, EmilieKovacs, Melinda HaydeeWidrlechner, MarkKovács, PéterWierzbowska, JadwigaKovalchuk, NinaWiewióra, BarbaraKovaleva, ValentinaWilkinson, Mark D.Kovanic, LudovitWilley, NeilKowalczewski, Przemysław ŁukaszWilliams, JamesKowalczyk, JanWillis, JonathanKowalczyk, MariuszWilson, Melissa L.Kowalewska, ŁucjaWiniarczyk, KrystynaKowalkowska, AgnieszkaWinkler, RobertKowalska, JolantaWińska, KatarzynaKowalski, RadosławWirtz, MarkusKozai, ToyokiWishart, JaneKozak, AnnaWiszniewska, AlinaKozak, BartoszWitkowicz, RobertKozaki, AkikoWitte, AngelaKozakiewicz, PawełWiwart, MarianKozieł, EdmundWłodarczak, SylwiaKozielewicz, PawelWłodarczyk, MaciejKozieradzka-Kiszkurno, MałgorzataWoch, Marcin W.Kozłowska, MariolaWójciak, MagdalenaKozma-Bognár, LászlóWojciechowska, NataliaKozyra, MałgorzataWojciechowska, RenataKrabel, DorisWójcik, Anna MariaKraj, WojciechWojcik, DanutaKrams, IndrikisWójcik-Gront, ElżbietaKraska, PiotrWójcikowska, BarbaraKraska, ThorstenWójcik-Pszczoła, KatarzynaKravchik, MichaelWojdylo, AnetaKremer, DarioWojtania, AgnieszkaKress, W. JohnWojtyla, ŁukaszKretschmer, MatthiasWolf, SebastianKrier, FrançoisWolna-Maruwka, AgnieszkaKrigas, NikosWolski, Grzegorz J.Krizman, MitjaWon, So YounKrólak, ElżbietaWong, AlanKrompiec, StanisławWong-Bajracharya, JohannaKrumova, SashkaWood, Delilah F.Krupa-Małkiewicz, MarcelinaWormit, AlexandraKruppert, SebastianWoźniak, GabrielaKruse, Lars H.Wozniak, KatarzynaKruszewska, JoannaWright, Carole LouiseKryštofová, SvetlanaWroniak, MałgorzataKryuchkov, FedorWu, DiKrzebietke, Sławomir JózefWu, Ding-taoKrzepiłko, AnnaWu, JiandongKrzyżak, JacekWu, Li-HsinKu, Kang-MoWu, LijunKubatka, PeterWu, LinboKubiak, DariuszWyszkowski, MirosławKubica, PawełXiao, JunKubicova, LenkaXiong, JiaKubisz, LeszekXofis, PanteleimonKubiszewski-Jakubiak, SzymonXu, HaoKubo, MiwaXylia, PanayiotaKubo, NakaoXynias, Ioannis Ν.Kučerová, AndreaYadav, DhananjayKucharski, JanYahia, MassinissaKucharski, MariuszYakolev, Igor A.Kuchitsu, KazuyukiYamada, MasashiKucinska, MalgorzataYamaguchi, Lydia FumikoKućko, AgataYan, HongxiangKudoyarova, Guzel R.Yáñez, RemediosKuhlmann, MarkusYang, Kai MinKujawski, RadosławYepez Gonzalez, Enrico ArturoKuklová, MargitaYin, ChuntaoKulczycki, GrzegorzYockteng, Roxana B.Kulhánek, MartinYokoi, ShujiKulig, BogdanYoo, Mi-jeongKulig, RyszardYoussef, Helmy M.Kulik, AnnaYu, BianyunKulikova, Natalya A.Yu, Chang YeonKulkarni, Krishnanand P.Yu, JingKulpa, DanutaYuan, PeiguoKulus, DariuszYudina, LyubovKumar, AjayYurchenko, EkaterinaKumar, AnujYuste-Lisbona, Fernando J.Kumar, ManojZafar, Syed AdeelKumar, ManuZagorchev, LyubenKumar, NarenderZaiter, AliKumar, PankajZaitlin, DavidKumar, RohitZambounis, AntoniosKume, AtsushiZammit Mangion, MarionKuna, LucijaZamocky, MarcelKundariya, HardikZamulina, InnaKunert, Karl J.Zanella, LorenzoKunwar, Ripu MardhanZanetti, StefaniaKurczyńska, EwaZarattini, MarcoKurek, MarcinZarei, ImanKurle, James E.Zarrelli, ArmandoKurousky, DmitriZarrouk, OlfaKurowska, MarzenaZawada, KatarzynaKus, PiotrZbigniew, MiszalskiKuśmierek-Tomaszewska, RenataZdarta, AgataKuśmierz, SebastianZebrowski, JacekKusstatscher, PeterZechmeister, HaraldKusunoki, KazutakaZeeshan, MuhammadKuzmanović, LjiljanaZeljkovic, CavarKuźniar, AgnieszkaZeljkovic, SanjaKwasniewska, JolantaZelnik, IgorKwiatkowska, DorotaZemetra, Robert S.Kwiecien, IngaZeng, ShaohuaKwon, Soon JaeZentella, RodolfoKwon, SoonwookZerbe, StefanKycko, MarlenaZhang, CaixiKyker, SarahZhang, CankuiKyraleou, MariaZhang, ChaozhongKyratzis, Angelos C.Zhang, JunliKyzioł, AgnieszkaZhao, MINGXIALa Spina, MichelangeloZhelev, PetarLabanca, FabianaZhou, JunŁabaz, BeataZhou, ManLabokas, JuozasZhou, MingxiLabuda, RomanZhou, RongLabudda, MateuszZhu, Qian-HaoLacaze, BernardZhuo, ChunliuLach, Michał StefanŽiarovská, JanaLachman, JaromirZielińska, SylwiaLācis, GunārsZienkiewicz, AgnieszkaLafite, PierreZienkiewicz, KrzysztofLahuta, Lesław BernardŽilius, ModestasLai, OlimpiaZimowska, BeataLaitung, BerylZoltner, MartinLakatos, TamásZou, ChengLake, Janice AnnZovko Končić, MarijanaLakomy, PiotrŻuk, MagdalenaLalak-Kańczugowska, JustynaZukiene, RasaLaloo, AndrewZuo, GuangleiLamade, EmmanuelleŻurek, GrzegorzLamari, FotiniZuzolo, DanielaLambardi, MaurizioŽvingila, DonatasLambrev, PetarZwart, RebeccaLambreva, Maya D.Żyżelewicz, DorotaLamponi, Stefania



